# Systematic screening identifies ABCG2 as critical factor underlying synergy of kinase inhibitors with transcriptional CDK inhibitors

**DOI:** 10.1186/s13058-023-01648-x

**Published:** 2023-05-05

**Authors:** Vera E. van der Noord, Wanda van der Stel, Gijs Louwerens, Danielle Verhoeven, Hendrik J. Kuiken, Cor Lieftink, Melanie Grandits, Gerhard F. Ecker, Roderick L. Beijersbergen, Peter Bouwman, Sylvia E. Le Dévédec, Bob van de Water

**Affiliations:** 1 Division of Drug Discovery and Safety, Leiden Academic Centre for Drug Research, Leiden University, Einsteinweg 55, 2333 CC Leiden, The Netherlands; 2 Division of Molecular Carcinogenesis, The NKI Robotics and Screening Center, The Netherlands Cancer Institute, Amsterdam, The Netherlands; 3 Department of Pharmaceutical Sciences, University of Vienna, Vienna, Austria

**Keywords:** Triple-negative breast cancer, Transcriptional cyclin-dependent kinases, CDK12/13, THZ531, ABCG2, Drug resistance

## Abstract

**Background:**

Triple-negative breast cancer (TNBC) is a subtype of breast cancer with limited treatment options and poor clinical prognosis. Inhibitors of transcriptional CDKs are currently under thorough investigation for application in the treatment of multiple cancer types, including breast cancer. These studies have raised interest in combining these inhibitors, including CDK12/13 inhibitor THZ531, with a variety of other anti-cancer agents. However, the full scope of these potential synergistic interactions of transcriptional CDK inhibitors with kinase inhibitors has not been systematically investigated. Moreover, the mechanisms behind these previously described synergistic interactions remain largely elusive.

**Methods:**

Kinase inhibitor combination screenings were performed to identify kinase inhibitors that synergize with CDK7 inhibitor THZ1 and CDK12/13 inhibitor THZ531 in TNBC cell lines. CRISPR-Cas9 knockout screening and transcriptomic evaluation of resistant versus sensitive cell lines were performed to identify genes critical for THZ531 resistance. RNA sequencing analysis after treatment with individual and combined synergistic treatments was performed to gain further insights into the mechanism of this synergy. Kinase inhibitor screening in combination with visualization of ABCG2-substrate pheophorbide A was used to identify kinase inhibitors that inhibit ABCG2. Multiple transcriptional CDK inhibitors were evaluated to extend the significance of the found mechanism to other transcriptional CDK inhibitors.

**Results:**

We show that a very high number of tyrosine kinase inhibitors synergize with the CDK12/13 inhibitor THZ531. Yet, we identified the multidrug transporter ABCG2 as key determinant of THZ531 resistance in TNBC cells. Mechanistically, we demonstrate that most synergistic kinase inhibitors block ABCG2 function, thereby sensitizing cells to transcriptional CDK inhibitors, including THZ531. Accordingly, these kinase inhibitors potentiate the effects of THZ531, disrupting gene expression and increasing intronic polyadenylation.

**Conclusion:**

Overall, this study demonstrates the critical role of ABCG2 in limiting the efficacy of transcriptional CDK inhibitors and identifies multiple kinase inhibitors that disrupt ABCG2 transporter function and thereby synergize with these CDK inhibitors. These findings therefore further facilitate the development of new (combination) therapies targeting transcriptional CDKs and highlight the importance of evaluating the role of ABC transporters in synergistic drug–drug interactions in general.

**Supplementary Information:**

The online version contains supplementary material available at 10.1186/s13058-023-01648-x.

## Background

Triple-negative breast cancer (TNBC) is an aggressive and heterogeneous subtype of breast cancer with poor prognosis, defined by lack of oestrogen receptor (ER), progesterone receptor (PR) and human epidermal growth factor receptor 2 (HER2) [1]. Targeted therapies against individual TNBC drivers (e.g. *MYC*, *PIK3CA* and *EGFR*) have not yet yielded clinical success, in part due to tumour heterogeneity and initial or acquired drug resistance.

Although inhibition of individual TNBC drivers that contribute to growth factor signalling pathways can easily be bypassed and lead to drug resistance, many of these pathways converge at one general point that is also targetable: transcriptional dysregulation facilitated by RNA polymerase II [2, 3]. Transcriptional cyclin-dependent kinases (CDKs) regulate RNA polymerase II activity by phosphorylating various residues of its C-terminal domain. While CDK7 mainly regulates transcriptional initiation, CDK9 and CDK12 regulate transcriptional elongation [4]. Moreover, CDK12 and CDK13 have been described to regulate transcriptional processing. However, the functions of these transcriptional CDKs are not limited to direct phosphorylation of RNA polymerase II and remain not fully understood. A notable consequence of CDK12/13 inactivation is the transcriptional downregulation of genes involved in DNA damage response and repair, resulting in a BRCAness phenotype that is synthetic lethal with PARP inhibition [5–8]. Importantly, inhibitors of transcriptional CDKs seem to specifically affect cancer cells, since the latter more strongly depend on transcriptional abnormalities such as super-enhancer- or MYC-driven transcription [3]. Multiple inhibitors of transcriptional CDKs have been evaluated in the preclinical setting as potential effective anti-cancer therapies in different cancer types, including breast cancer [9]. Moreover, we have previously observed that CDC7/CDK9 inhibition by PHA-767491 can be potentiated by combination therapy with EGFR inhibitors, including lapatinib [10]. In addition, multiple other studies have proposed synergistic combinations of transcriptional CDK inhibitors with a variety of other inhibitors in addition to EGFR/HER2 inhibitors, for example Raf, Bcl-2 and PARP inhibitors [11–15]. The proposed mechanisms behind these synergistic interactions are diverse and not entirely clear. While some studies suggest that CDK inhibitors prevent adaptive, pro-survival responses [11–13, 15–17], others propose that CDK inhibitors may interfere with the specific pathways that are targeted by other drugs [14, 18–21].

In this study, we systematically investigate the synergy of kinase inhibitors with transcriptional CDK inhibitors in TNBC cell lines. Using kinase inhibitor screens, we reveal that a strikingly large number of kinase inhibitors synergize with the CDK12/13 inhibitor THZ531 and inhibit ABCG2 transporter activity. Importantly, genome-wide CRISPR-Cas9 knockout screening suggests that ABCG2 inhibition is the major driving force behind the synergistic interactions with THZ531 and other transcriptional CDK inhibitors in TNBC.

## Methods

### Cell culture

All TNBC cell lines and ER + breast cancer cells were provided by Prof. J. Martens (Erasmus MC, the Netherlands). Cell lines were cultured in RPMI-1640 medium containing L-glutamine and 25 mM HEPES (Gibco, Fisher Scientific) supplemented with 10% foetal bovine serum, 25 U/mL penicillin, and 25 µg/mL streptomycin (Fisher Scientific) in a humidified incubator with 5% CO_2_ at 37 °C.

### Reagents and antibodies

For combination screening of kinase inhibitors with CDK inhibitors, a 378-kinase inhibitor library (L1200, Selleckchem) was used at 1 µM. For screening of ABCG2 activity using pheophorbide A, an updated batch of this library with 760 kinase inhibitors (363 overlapping inhibitors with previous library) was screened at 1 µM.

PHA-767491 (S2742), lapatinib (S2111), erlotinib (S7786) and flavopiridol (S1230) were from Selleckchem. CDKI-73 was provided by Prof. S. Wang (University of South-Australia) [22]. BAY-143572 (HY-12871B), THZ1 (HY-80013A), THZ531 (HY-103618), SR4835 (HY-130250), nilotinib (HY-10159), rabusertib (LY2603618, HY-14720) were purchased from MedChemExpress. Momelotinib (CYT387, S2219) and ralimetinib (LY2228820, S1494) were used from the Selleckchem L1200 kinase inhibitor library. Cisplatin was from the Leiden University Medical Center pharmacy (Leiden, The Netherlands).

Mouse RNA-polymerase II (RPB1 CTD) antibody (#2629) and rabbit MEK1/2 (#8727), phospho-MEK1/2 (Ser217/221, #9154), Akt (#9272), phospho-AKT (Ser473, #9271), ERK1/2 (#4695), phospho-ERK1/2 (Thr202/Tyr204, #9101), MCL-1 (#5453), phospho-RNA polymerase II (Ser2, #13499), phospho-RNA polymerase II (Ser5, #13,523), total PARP (#9542) and cleaved PARP (Asp214, #9541) were from Cell Signalling Technology. The mouse antibody against α-tubulin (#T-9026) was purchased from Sigma-Aldrich. Horseradish peroxidase (HRP)-linked anti-rabbit (#111-035-003) and anti-mouse (#115-035-003) and Alexa-647-linked anti-mouse (#115-605-146) secondary goat antibodies were from Jackson Immunoresearch.

### Cell proliferation assays and definition of synergy

Cell proliferation was evaluated after 4 days of treatment using sulforhodamine B (SRB) colorimetric assay [23]. TNBC cell lines were plated in 96-well plates and cells were treated with inhibitors the next day. After 4 days of treatment, cells were fixed by addition of 50% trichloroacetic acid (TCA, 30 µL in 100 µL medium, Sigma-Aldrich). Cells were stained with 0.4% SRB (Sigma-Aldrich) in 1% acetic acid (VWR) and washed 4X with 1% acetic acid to remove unbound SRB. Bound SRB was solubilized in 10 mM unbuffered Tris (Fischer Scientific) and SRB absorbance (540 nm) was measured after 2 h on a Tecan Infinite M1000 microplate reader. Dose–response curves and IC50 values were generated in GraphPad Prism (version 9). The percentage point of synergy was calculated as the difference between combined responses and the summed or additive response of each inhibitor as single treatment. Bliss and Loewe synergy scores were calculated using the SynergyFinderPlus web application [24]. Synergy scores for individual concentrations higher than 10 are generally considered synergistic. The mean of these individual synergy scores across the entire dose range was used for comparison of extent of synergy between different cell lines or conditions. Here, we considered combination treatments with a mean Bliss/Loewe synergy score > 20 as strongly synergistic.

### Protein extraction and western blotting

Proteins were extracted, and western blotting was performed, using SDS-PAGE, as described previously [25]. Briefly, proteins were extracted using RIPA buffer and cellular proteins were denatured in soluble protein buffer containing 2-mercaptoethanol (final concentration 1.7%). Proteins (20 µg/lane) were loaded into 7.5% polyacrylamide gels for SDS-PAGE. Proteins were transferred to polyvinylidene fluoride (PVDF) membranes (Merck) and blocked with 5% bovine serum albumin (BSA; Sigma-Aldrich) in Tris-buffered saline with 0.05% Tween-20 (TBS-T_0.05_). Membranes were subsequently incubated with primary and secondary antibodies in 1% BSA in TBS-T_0.05_. Prior to imaging, HRP-conjugated secondary antibodies were incubated with ECL Prime Western Blotting Detection Reagent (GE Healthcare Life Sciences) for 3 min. Chemiluminescence or fluorescence was detected on an Amersham Imager 600 (GE Healthcare Life Sciences). Uncropped images used of western blots are available in Additional file Figures (Additional File 1: Fig. S12).

### FUCCI cell cycle and AnnexinV/PI cell death assay

FUCCI plasmid pLL3.7 m-Clover-Geminin(1-110)-IRES-mKO2-Cdt(30-120) was a gift from Michael Lin (Addgene plasmid #83841) [26] and was used for lentiviral transduction of Hs578T cells. Hs578T cells expressing the construct were selected using FACS cell sorting.

For both AnnexinV/PI cell death and FUCCI cell cycle analysis, cells were seeded in µclear 96-wells black-bottom imaging plates (#655090, Corning). The next day, cells were stained with Hoechst-33342 (100 ng/mL) and then treated with inhibitors. For AnnexinV/PI assays, Annexin V-Alexa633 (0.05%) and PI (100 nM) were added to the cells simultaneously with the inhibitors. Live cell imaging of FUCCI cell cycle indicators and AnnexinV/PI signals was performed at indicated timepoints using a 10X objective on a ImageXpress Micro XLS imager (Molecular Devices). Images were analysed in Cell Profiler, and percentage of positive cells from total number of cells were reported. For FUCCI cell cycle analysis, we defined Cdt1-positive cells as cells in G1 phase, Cdt1- and Geminin-positive cells as cells in the G1/S transition, Geminin-positive cells as cells in S, G2 or M-phase and negative cells as cells in transition from M to G1 phase [26].

### Small interfering RNA (siRNA) knockdown

siRNAs were from the human siGENOME libraries (Dharmacon). siRNA knockdown was performed by reverse transfection with 25 nM SMARTpool siRNAs using INTERFERin transfection reagent (Polyplus, 409-50). Medium was refreshed after 18 h. 48 h after transfection, cells were treated with the inhibitors for 4 days. Day 0 plates for SRB assays were fixated on day of treatment. Controls were cells treated with transfection reagent only (mock) or a mixture of 720 siRNAs targeting different kinase genes, thereby not significantly affecting any gene expression (non-specific kinase pool, siKP). Knockdown data were normalized to siKP and day 0, unless stated otherwise.

### CRISPR-Cas9 knockout

Hs578T, SKBR7 and MDA-MB-231 with inducible Cas9 and gRNA’s were generated by lentiviral transduction with the Edit-R inducible lentiviral Cas9 plasmid (Dharmacon) and sgRNAs. Non-targeting control and sgRNAs against CDK12 and ABCG2 were from the human Sanger Arrayed Whole Genome Lentiviral CRISPR Library (Sigma–Aldrich) and were provided by Prof. R. Hoeben (Leiden University Medical Centre, The Netherlands). Cells were selected using puromycin (1 µg/ml). Knockouts were generated by induction of Cas9 expression with doxycycline (1 µg/mL) for 3 days. Knockout efficiency was evaluated after 4 days using TIDE analysis, as described previously [27] and/or Western blotting. For proliferation assays, cells were re-seeded 3 days after doxycycline induction and were incubated the next day with the inhibitors for an additional 4 days.

### CRISPR Cas9 knockout screen

Hs578T cells with inducible Cas9 were transduced with the Brunello pooled human CRISPR knockout library (#73178, Addgene) at a multiplicity of infection of around 0.1. Following 5 days of selection with puromycin (1 µg/mL) and 7 days of doxycycline-induced expression of Cas9, reference samples were collected (*t* = 0) and cells were separated into different treatment arms with three independent replicates each. Cells were cultured in the presence of THZ531 (0.1 µM) or DMSO (control) while maintaining at least 20 million cells at all times, ensuring a 250X coverage of the library throughout all steps. After both arms reached at least 8 population doublings, cells were collected and stored as pellets at -80ºC.

Genomic DNA was isolated using the Gentra^®^ Puregene^®^ kit (#158767, Qiagen) following the manufacture’s protocol specified for cultured cells and dissolved in the hydration solution overnight while shaking at room temperature. DNA yield ranged between 276 and 384 µg per sample. The genomic DNA was divided into multiple reactions per sample (50 µg each, using all material) and fragmented at 37 °C overnight, using 50 U Ndel enzyme (R0111L), 50U Pstl-HF enzyme (R3140L), and 50 µL 10X cutSmart^®^ buffer (B7204S) from New England Biolabs, supplemented to 500 µL with nuclease-free water (AM9932, Thermo Fisher). The reactions were heated to 100 °C for 10 min., and following addition of 500 µL 2 M NaCl, reheated to 100 °C for 5 min. and then immediately snap-frozen in liquid nitrogen. Per tube and prior to thawing, 1 µL of each 10 µM 5′ biotinylated capture oligo (TGCTTACCGTAACTTGAAAGTATTTCGATTTCTTGGCTTTATATATCTTG and TGCAGCCAGGTGGAAGTAATTCAAGGCACGCAAGGGCCATAACCCGTAAA) was added on top of the frozen solution, which was then immediately transferred to a thermoshaker for overnight hybridization at 60 °C. Next, to capture hybridized DNA, 20 µL Streptavidin T1 Dynabeads (#65602, ThermoFisher) were washed three times with 500 µL wash buffer (1 M NaCl, 10 mM Tris–HCl, pH 8), added to each tube, and incubated under rotation at room temperature for 2 h. The beads were washed twice with wash buffer and twice with 10 mM Tris–HCl (pH 8). Non-hybridized biotinylated oligos were digested in 50 µL reactions composed of 44 µL 10 mM Tris–HCl (pH 8), 5 µL 10X Exonuclease buffer, and 1 µL Exonuclease I (M0293L, New England Biolabs), at 37 °C for 1 h. Beads were washed 3 times with 10 mM Tris–HCl (pH 8) and resuspended in 20 µL 10 mM Tris–HCl (pH 8). Two rounds of PCR were performed to amplify the gRNA sequences. In the first PCR, distinct forward primers that each encode a unique barcode sequence and facilitate deconvolution of sequence reads of pooled samples (ACACTCTTTCCCTACACGACGCTCTTCCGATCTNNNNNNGGCTTTATATATCTTGTGGAAAGGACG with NNNNNN representing barcode sequences ACATCG, TGGTCA, CACTGT, ATTGGC, GATCTG, TCAAGT, and TACAAG) were used in combination with a common reverse primer (GTGACTGGAGTTCAGACGTGTGCTCTTCCGATCTTCTACTATTCTTTCCCCTGCACTGT). PCR mixture: 1 μL 10 μM forward primer, 1 μL 10 μM reverse primer, 1 μL 10 mM dNTPs (R0193, ThermoFisher), 0.5 μL Phusion polymerase and 10 μL 5X HF buffer (M0530L, New England Biolabs), supplemented with nuclease-free water to a total volume of 50 μL. PCR cycling conditions: 3 min. @ 98 °C, 20 times (30 s. @ 98 °C, 30 s. @ 60 °C, 30 s. @ 72 °C), and 5 min @ 72 °C. Per sample, products of individual reactions were pooled and 2 μL of each pool was used as template in the second PCR with conditions similar to the first, but having 15 instead of 20 cycles, to add the p5 and p7 adapter sequences (primers: AATGATACGGCGACCACCGAGATCTACACTCTTTCCCTACACGACGCTCTTCCGATCT and CAAGCAGAAGACGGCATACGAGATATCACGGTGACTGGAGTTCAGACGTGTGCTCTTCCGATCT). The PCR products were purified using the Bioline ISOLATE II PCR and Gel kit (BIO-52060, GC biotech) following the manufacture’s protocol, subjected to quality control assays (DNA concentration and purity, fragment size by gel electrophoreses), and subsequently pooled by combining 150 ng of each sample. The pool was sequenced on a Illumina HiSeq 2500, and the reads were mapped to the unique barcodes used for each sample and the Brunello library. Differential analysis on the single sgRNA level was performed using DESeq2 [28]. Subsequently, the MAGeCK ranking aggregation method was used to prioritize sgRNAs and calculate *p* values for each gene, which were corrected for multiple testing using the Benjamini–Hochberg method [29].

### Gene set enrichment analysis (GSEA) of TNBC cell lines

TNBC cell line mRNA microarray (GSE41313) and RNA sequencing data were previously established [30, 31]. Data were log-transformed (log_2_), and rank-ordered genes (signal2Noise) were evaluated using GSEA software. Cell lines were grouped as either THZ531-sensitive (IC50 < 0.1 µM) and non-synergistic (either Bliss/Loewe synergy score lapatinib or nilotinib < 10) or THZ531-resistant (IC50 > 0.1 µM) and synergistic (both Bliss/Loewe synergy score lapatinib and nilotinib > 20) and used for phenotype analysis in GSEA. Cell lines that did not meet these conditions (MDA-MB-231, HCC1143, HCC1806) were excluded from GSEA analysis.

### Pheophorbide A accumulation

Cells were incubated with inhibitors and pheophorbide A (0.25 µM) for 23 h. 1 h prior to imaging, cells were stained with Hoechst-33342 (100 ng/mL) by directly adding this in the medium. Cells were imaged on a Nikon Eclipse TiE2000 confocal microscope using 20 × Plan Apo objective 24 h after treatment. Images were analysed in Cell Profiler. Normalized pheophorbide A intensity was calculated by dividing the total pheophorbide A intensity by the number of nuclei, and this value was normalized to DMSO. For kinase inhibitor screening a normalized total pheophorbide A intensity of > 1.2 in both replicates was considered as pheophorbide A accumulation. Kinase inhibitors that had a normalized intensity of > 1.2 in one of the two independent experiments, but < 1.2 in the other, were classified as “uncertain”. Normalized pheophorbide A accumulation was correlated to extent of synergy for kinase inhibitors with THZ531 only for kinase inhibitors that did not strongly affect proliferation as single treatment (> 40% proliferation compared to DMSO).

### ABCG2 inhibitory activity predictive modelling

The dataset used for the training of the classification model of ABCG2 inhibition is based on previously published data [32]. This original data set was used to generate a prediction model based on a logistic regression algorithm and is freely available for the scientific community as a web service (https://livertox.univie.ac.at) [33]. The dataset used in the current study was further enriched by including compounds obtained from the open-source databases ChEMBL and PubChem. The retrieved compounds were standardized using a modified Atkinson standardization protocol (available at github.com/flatkinson/standardiser). In more detail, bonds to alkali metals and alkaline earth metals were removed. In case of a mixture, the fragments were split, and each fragment was standardized separately. Non-organic compounds were eliminated, and functional groups were neutralized. Duplicates were removed from the dataset including stereoisomers. Further, if duplicate compounds between different sources disagree in the classification label, they were excluded. Compounds with an activity value below 10 µM were labelled as an inhibitor and above this threshold as non-inhibitor. The acquired data set contained in total 1442 compounds, which was divided into 776 inhibitors and 666 non-inhibitors. The performance of the model was validated by a tenfold cross-validation as well as an external test set. This test set was obtained by excluding 10% of the original dataset using the sample method implemented in pandas with a random state of 0. Finally, 1298 compounds (694 inhibitors and 604 non-inhibitors) were used for the training and 144 compounds (82 inhibitors and 62 non-inhibitors) for the validation step.

For the chemical representation of the compounds, molecular descriptors as implemented in RDKit (version 2019.03.3) were used. For the training of the prediction model, a random forest classifier was chosen based on the scikit-learn [34] (version 0.21.2) libraries. The model showed an accuracy of 0.85, which corresponds to the rate of correct predictions. Eighty-five percentage of actual positives were correctly identified as well as 85% of actual negatives, which is indicated by the sensitivity and specificity. Further, the Matthews correlation coefficient (MCC) was determined to estimate the quality of the models. MCC differentiates between random and well performing predictions. While a value of 0 indicates that a prediction is just random, a value of 1 indicates a perfect prediction and − 1 a wrong prediction. The score for the classification model of ABCG2 activity is 0.70. For the external test set, the sensitivity was comparable with the tenfold cross-validation with a value of 0.84 and slightly lower for the specificity with a value of 0.76.

Kinase inhibitors that had a ABCG2 inhibitory prediction score ≥ 0.7 were classified as “active”, while kinase inhibitors with a score of 0.4–0.6 were classified as "weak or uncertain” and kinase inhibitors with a score ≤ 0.3 were classified as “inactive”.

### RNA sequencing and analysis

RNA was isolated from Hs578T cells 8 h after treatment with the inhibitors using the RNeasy Plus Mini Kit (Qiagen). RNA quality and quantity was measured on an Agilent-4200 Bioanalyzer. Unstranded PolyA-selected libraries were prepared using the DNBseq platform, and 50 M 100-bp paired-end reads were sequenced on a DNBSEQ-G400 sequencer by BGI Europe. Filtered reads were aligned to Grch38 using Hisat2 [35]. For differential gene expression analysis, aligned reads were counted using featureCounts [36]. Normalization and differential gene expression analysis were performed using DESeq2 [28]. Pathway enrichment of ranked gene lists was evaluated in STRING [37].

For quantification of intronic polyadenylation (IPA), the PolyAsite atlas (version 2.0), a database of genomic locations of alternative polyadenylation sites previously determined by 3′ end sequencing, was used [38]. To evaluate IPA events, we used custom scripts to quantify differential exon usage after IPA sites and expression of IPA sites (scripts available upon request). Briefly, intronic reads spanning − 10 and + 10 basepairs of intronic polyadenylation sites (exonic reads excluded) were counted from the aligned reads of the RNA sequencing data using HTSeq-count and custom GTF files describing these regions and exons [39]. Log2 fold changes and adjusted *p* values of exons and IPA sites were quantified using DESeq2. Upregulation of IPA sites was considered significant for log2 fold changes > 1 and adjusted *p* values < 0.001. Stringent *p* values and minimum counts of 10 reads for a minimum of 3 samples were used to exclude falsely increased IPA usage due to noise in intronic regions.

Exon usage was quantified using DEXSeq-count [40]. Counts of exons before (upstream of), and after (downstream of) IPA sites of an individual gene were summed, creating overall exon counts from the part of the gene before and after the IPA site. These were normalized and quantified using DESeq2. Gene parts downstream and upstream of the IPA site were considered to be significantly differentially used when the difference in log2 fold change downstream and upstream of the IPA site was larger than 0.75 and the part downstream of the IPA site was significantly altered with adjusted *p* value < 0.01. IPA sites significantly upregulated and resulting in differentially expressed gene parts were considered significant IPA events.

### Statistical analysis and data visualization

Statistical tests were performed in GraphPad Prism and are further specified in corresponding figure legends. For all figures, significance was expressed as follows: *p < 0.05, **p < 0.01, ***p < 0.001, ****p < 0.0001.

## Results

### CDK12/13 inhibitor THZ531 strongly synergizes with tyrosine kinase inhibitors

**Fig. 1:**
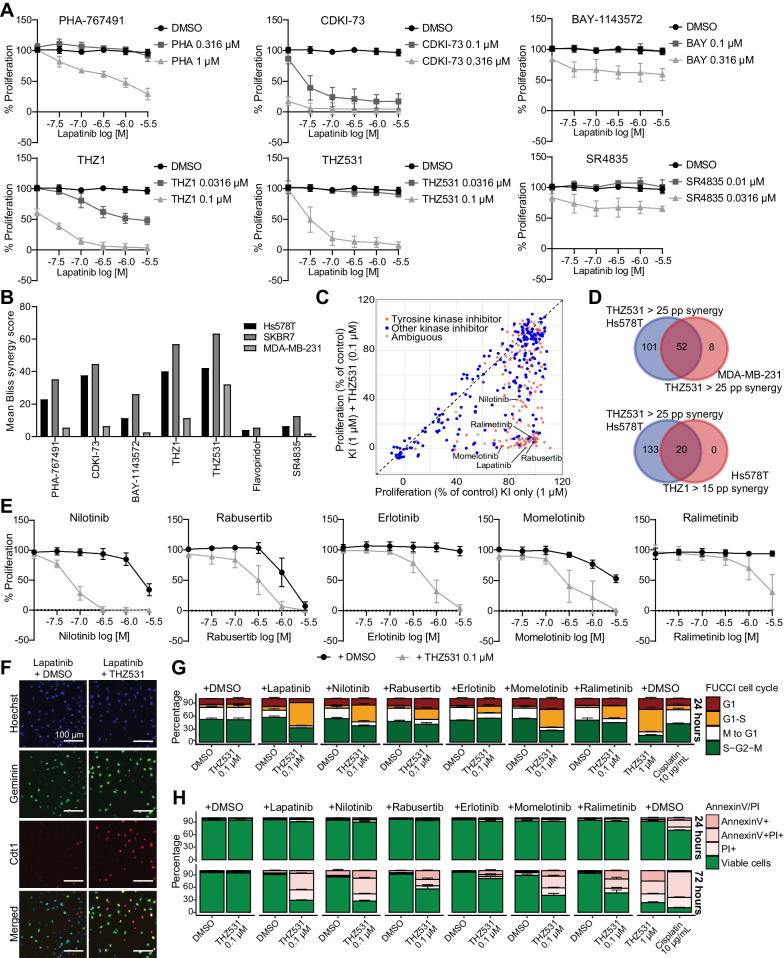
Tyrosine kinase inhibitors synergize with THZ531 to halt TNBC proliferation. **A** Proliferative response upon treatment with dose range of lapatinib in combination with transcriptional CDK inhibitors PHA-767491 (0.316 and 1 µM), CDKI-73 (0.1 and 0.316 µM), BAY-1143572 (0.1 and 0.316 µM), THZ1 (0.0316 and 0.1 µM), THZ531 (0.0316 and 0.1 µM) and SR4835 (0.01 and 0.0316 µM) in Hs578T. Data are the mean (± SD) of three independent experiments. **B** Corresponding mean bliss synergy scores of these CDK inhibitors with lapatinib, in Hs578T, SKBR7 and MDA-MB-231. **C** Proliferative responses in kinase inhibitor (KI) screening upon treatment with tyrosine (red) or other (blue) KI’s only (1 µM) or in combination with THZ531 (0.1 µM) in Hs578T. Data are the mean of two independent experiments. **D** Overlap between synergistic hits with more than 25 percentage point (pp) difference between the combined and additive effects of THZ531 (0.1 µM) and kinase inhibitors (1 µM) in Hs578T and MDA-MB-231 (upper). Overlap between synergistic hits in combination treatment with THZ531 (0.1 µM, 25 pp synergy) and THZ1 (0.0316 µM, 15 pp synergy) (lower). **E** Proliferation upon treatment with dose ranges of nilotinib, rabusertib, erlotinib, momelotinib, ralimetinib alone or in combination with THZ531 (0.1 µM) in Hs578T. Data are the mean (± SD) of four independent experiments. **F, G** Representative images **(F)** and percentages **(G)** of FUCCI cell cycle progression 24 h after treatment with lapatinib (3.16 µM), nilotinib, rabusertib, erlotinib, momelotinib and ralimetinib (all 1 µM) alone or in combination with THZ531 (0.1 µM) and single treatment of high dose THZ531 (1 µM) or cisplatin (10 µg/mL). **H** Induction of (apoptotic) cell death measured by Annexin V and PI staining after 24 h and 72 h of treatment with these inhibitors. Cell death and FUCCI data are the mean (± SD) of three independent experiments

We have previously demonstrated that the HER2/EGFR inhibitor lapatinib synergizes with the CDC7/CDK9 inhibitor PHA-767491 in TNBC cells [10]. To investigate whether this is also the case for other transcriptional CDK inhibitors, we treated the TNBC cell lines Hs578T, SKBR7 and MDA-MB-231 with a combination of lapatinib and CDC7/CDK9 inhibitor PHA-767491, CDK9 inhibitor CDKI-73, CDK9 inhibitor BAY-1143572, CDK7 inhibitor THZ1, CDK12/13 inhibitor THZ531 or CDK12/13 inhibitor SR4835. In addition to PHA-767491, lapatinib synergized most strongly with CDKI-73, THZ1 and THZ531 in Hs578T, SKBR7 and MDA-MB-231 cells (Fig. 1A and B, Additional file 1: Figure S1A–D). However, the combination of lapatinib with SR-4835, BAY-1143572, or flavopiridol did not induce a similarly strong effect in all of these cell lines.

Given the relatively high synergy scores for THZ1 and THZ531 and the fact that several studies demonstrated synergistic activity with other compounds [11, 13, 18–20], we aimed to systematically assess the kinase inhibitor landscape that could sensitize TNBC to these CDK inhibitors. We therefore screened a combination of THZ531 or THZ1 with 378 kinase inhibitors in Hs578T and MDA-MB-231 TNBC cells. We found that a strikingly large number of kinase inhibitors synergized with THZ531 in Hs578T (153 kinase inhibitors with > 25 percentage point of synergy) and MDA-MB-231 cells (60 kinase inhibitors with > 25 percentage point of synergy) (Fig. 1C and D, Additional file 1: Fig. S2A; Additional file 2: Table S1). A smaller number of kinase inhibitors synergized with THZ1 in Hs578T cells (20 kinase inhibitors with > 15 percentage point of synergy), all of which also synergized with THZ531 (Fig. 1D, Additional file 1: Fig. S2B; Additional file 2: Table S1), suggesting a similar underlying mechanism. In MDA-MB-231 cells, THZ1 also synergized with many kinase inhibitors (58 kinase inhibitors with > 25 percentage point of synergy), although single treatment of THZ1 at this concentration already affected their proliferation slightly (Additional file 1: Fig. S2B; Additional file 2: Table S1). Although the kinase inhibitors that synergized with THZ531 have a wide range of targets across multiple pathways, inhibitors targeting (receptor) tyrosine kinases induced the most synergy with THZ531 (Fig. 1C, Additional file 1: Fig. S2C, *p* < 0.0001).

For further validation and exploration of the synergistic drug interactions, we selected several compounds, currently in clinical trials or FDA approved, that displayed more than 25 percentage point of synergy in both Hs578T and MDA-MB-231 cells and that inhibit different targets, including EGFR tyrosine kinase inhibitors lapatinib and erlotinib, Bcr-Abl tyrosine kinase inhibitor nilotinib, Chk1/2 serine/threonine inhibitor rabusertib, JAK1/2 tyrosine kinase inhibitor momelotinib and p38 MAPK serine/threonine kinase inhibitor ralimetinib. These compounds indeed induced synergy with THZ531 across a wide range of concentrations in TNBC cell lines Hs578T (Fig. 1E), SKBR7 (Additional file 1: Fig. S2D) and MDA-MB-231 (Additional file 1: Fig. S2E). Altogether, these data demonstrate that THZ531 strongly synergizes with a large variety of kinase inhibitors.

### Combination treatment with kinase inhibitors and THZ531 halts cells in G1/S checkpoint and causes cell death

Next, we further investigated the mechanism behind the growth inhibition upon the combination treatments with THZ531. Therefore, we assessed the cell cycle progression using Hs578T cells expressing the fluorescent cell cycle indicators (FUCCI) [26]. Whilst the single treatments did not affect cell cycle progression, the combination treatments with THZ531 (0.1 µM) and lapatinib, nilotinib, rabusertib, momelotinib or ralimetinib synergistically halted Hs578T cells in the G1/S checkpoint, already within 12 h of combination treatment, which became more prominent after 24 h of treatment (Fig. 1F and G, and Additional file 1: Fig. S3A). In addition, using Annexin V and propidium iodide (PI) staining, we studied whether this combination treatment only arrests cells in the cell cycle, or whether it eventually also kills TNBC cells. Indeed, at 48 and 72 h, but not yet at 24 h, these combination treatments synergistically induced apoptotic cell death in Hs578T and SKBR7 cells (Fig. 1I, Additional file 1: Fig. S3B and C). Of note, the cell death and cell cycle arrest dynamics induced by the combination treatments were similar to treatment of THZ531 at tenfold higher dosage (1 µM). Together these data demonstrate that the combination of THZ531 and a diverse set of kinase inhibitors halts Hs578T cells in G1/S checkpoint and subsequently causes cell death.

### THZ531 resistance is associated with synergy between THZ531 and kinase inhibitors and predominantly dependent on ABCG2

**Fig. 2:**
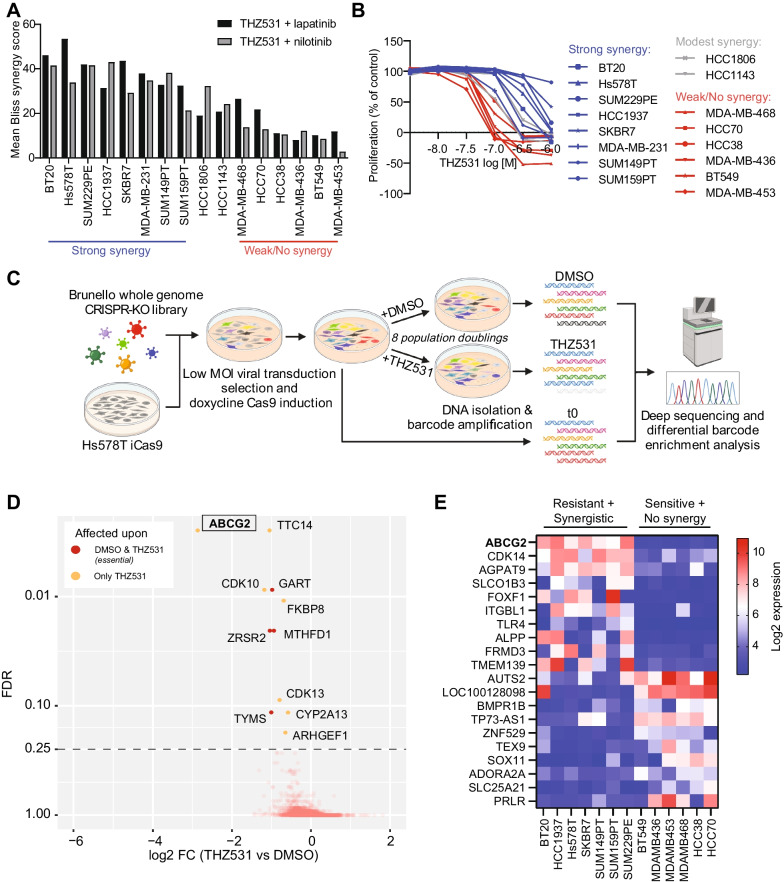
ABCG2 is associated with drug resistance and synergy with THZ531. **A** Bliss synergy scores for a panel of TNBC cell lines treated with a dose range of lapatinib and nilotinib, in combination with THZ531 0.0316 and 0.1 µM. Data are the mean (± SD) of two (MDA-MB-468, MDA-MB-436 and SUM159PT) or three (other cell lines) independent experiments. **B** Proliferative responses of TNBC cell line panel to a dose range of THZ531 alone. Lines are coloured by extent of synergy with lapatinib and nilotinib and THZ531 (blue, strong synergy; grey, modest synergy; red, weak synergy) **C** Schematic overview of CRISPR-Cas9 dropout screen for genes that sensitize Hs578T cells to THZ531 0.1 µM. This illustration was created with BioRender.com. **D** CRISPR-Cas9 dropout screening results showing median Log2 FC and corresponding FDR *p* value of gRNA levels in in Hs578T THZ531-treated pool versus DMSO. Orange dots present gene knockouts that were only affected upon THZ531 treatment, while red dots indicate gene knockouts that were also reduced in DMSO compared to T0. Data show the mean from three independent experiments and use the median Log2 FC of multiple different sgRNA’s per gene. **E** Rank-ordered microarray-based mRNA expression from gene set enrichment analysis of THZ531-resistant and synergistic cell lines versus THZ531-sensitive and non-synergistic cell lines showing the top and bottom 10 genes correlated with this phenotype

To further examine the relevance of these synergistic interactions, we investigated whether they also apply to other TNBC cell lines. Indeed, the combination of THZ531 and lapatinib or nilotinib also synergistically inhibited cell proliferation in BT20, SUM229PE, SUM149PT, HCC1937 and SUM159PT TNBC cell lines (Bliss and Loewe synergy scores for lapatinib and nilotinib combinations > 20) (Fig. 2A, Additional file 1: Figure S4). Lapatinib or nilotinib also sensitized HCC1143 and HCC1806 cells to THZ531, albeit to a lesser extent (Bliss or Loewe synergy score of lapatinib or nilotinib between 10 and 20), while in MDA-MB-468, MDA-MB-436, BT549, HCC38, MDA-MB-453 and HCC70 the synergy was limited (Bliss or Loewe score of lapatinib or nilotinib < 10). Of note, cell lines that were sensitized to THZ531 by lapatinib or nilotinib were relatively resistant (IC50 ranging from 0.2 to 4.6 µM) to THZ531 treatment alone, while cell lines that did not demonstrate strong synergy were relatively sensitive (IC50 ranging from 0.032 to 0.1 µM) to THZ531 (Fig. 2B). This observation was not specific for TNBC cells, as it also applied to ER + cell lines MCF7 and T47D (Additional file 1: Fig. S4).

As THZ531 sensitivity was associated with the extend of synergy with tyrosine kinase inhibitors, we further investigated the mechanisms of resistance to THZ531 in TNBC cells. Using a whole genome pooled CRISPR/Cas9 knockout screen, we identified 11 genes that support the proliferation of Hs578T cells in the presence of THZ531 (Fig. 2C). Knockout of *ABCG2*, *TTC14*, *CDK10*, *FKBP8*, *CDK13*, *CYP2A13* and *ARHGEF1* only affected proliferation of Hs578T cells in the presence of THZ531, with *ABCG2* being the strongest regulator of sensitivity (Fig. 2D, Additional file 1: Fig. S5A).

We also investigated which transcriptomic features distinguish the cell lines resistant to THZ531, and demonstrating synergy in combination with lapatinib or nilotinib, from the cell lines sensitive to THZ531 and not demonstrating this synergy. For this purpose, we utilized previously established microarray and RNA sequencing-based expression data [30, 31]. Both analyses pointed out a high expression of ABCG2 in the cell lines demonstrating the resistant and synergistic phenotype (Fig. 2E, Additional file 1: Fig. S5B). Importantly, previous studies have shown that multi-drug ABC transporters ABCB1 or ABCG2 can cause acquired resistance to THZ1 upon long-term drug selection by lowering intracellular THZ1 concentrations [41, 42] and that this can cause cross-resistance to THZ531 [41]. Notably, ABCG2 was the only gene associated with the resistant and synergistic phenotype that overlapped with the significant hits in the CRISPR-Cas9 knockout screen. Moreover, the genes identified by the CRISPR-Cas9 knockout screen and phenotypic associations do not overlap with any of the intended targets of the synergistic kinase inhibitors, suggesting that these inhibitors may sensitize the cells to THZ531 by directly interacting with one of the identified proteins, most likely ABCG2 (Additional file 1: Fig. S5C; Additional file 3: Table S2). Consistent with this hypothesis, multiple tyrosine kinase inhibitors, including lapatinib and nilotinib, have been previously identified to also inhibit ABCG2 [43]. To further rule out target-related mechanisms of synergy, we investigated whether lapatinib or nilotinib sensitized cells to depletion of CDK12 and CDK13. *CDK12* knockout in Hs578T or MDA-MB-231 cells or knockdown of *CDK12* and/or *CDK13* in Hs578T cells did not increase sensitivity to lapatinib or nilotinib (Additional file 1: Fig. S5D–F).

### ABCG2 inhibition mimics synergistic effects of kinase inhibitors with transcriptional CDK inhibitors

**Fig. 3:**
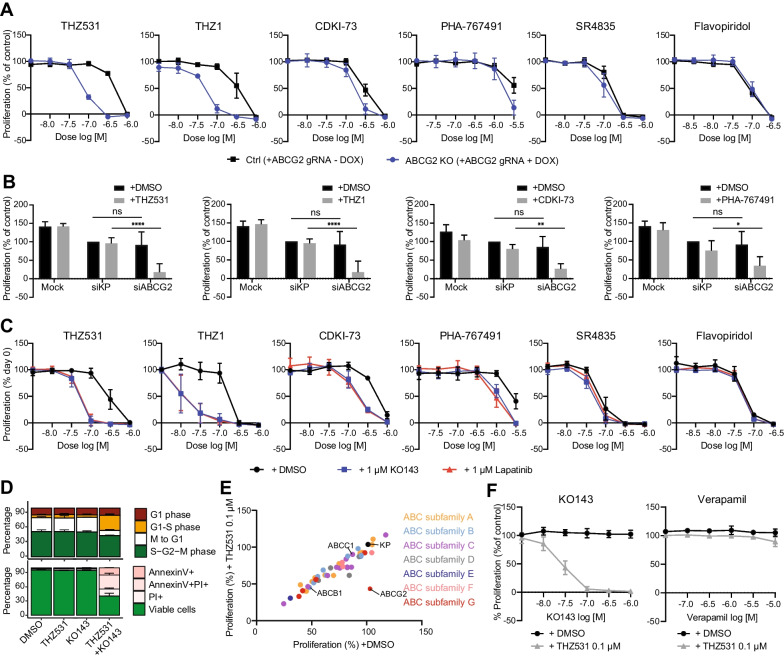
ABCG2 inhibition and depletion synergizes with THZ531 in similar fashion as synergistic kinase inhibitors. **A** Proliferative responses of dose ranges of transcriptional CDK inhibitors THZ531, THZ1, CDKI-73, PHA-767491, SR4835 and flavopiridol in control cells (Hs578T with inducible Cas9 and ABCG2 gRNA, without addition of doxycycline) or ABCG2 knockout cells (with addition of doxycycline). **B** Proliferative response to THZ531 (0.1 µM), THZ1 (0.0316 µM), CDKI-73 (0.1 µM), PHA-767491 (1 µM) in untransfected (mock) Hs578T cells or Hs578T cells treated with siKP (ctrl) or siABCG2. **C** Proliferative responses of dose ranges of transcriptional CDK inhibitors THZ531, THZ1, CDKI-73, PHA-767491, SR4835 and flavopiridol, alone, or in combination with KO143 (1 µM) or lapatinib (1 µM). **D** Effects of combination treatment of KO143 (1 µM) with THZ531 (0.1 µM) on FUCCI cell cycle (upper, 24 h) and cell death AnnexinV/PI (lower, 72 h) in Hs578T cells. **E** ABC-transporter siRNA screen showing proliferative responses upon knockdown of ABC-transporter genes together with DMSO or THZ531 (0.1 µM) treatment. Means of DMSO or treatment were compared using multiple comparisons (Dunnet’s correction) from two-way ANOVA between siKP and siABCG2. **F** Proliferative responses of combination treatment of a dose range of either ABCG2 inhibitor KO143 or ABCB1/ABCC1 inhibitor Verapamil with THZ531 0.1 µM. All data are the mean (± SD) from three independent experiments

We next investigated whether the role of ABCG2 in resistance to THZ531 in Hs578T cells extends to the other CDK inhibitors that synergized with lapatinib, and whether this also holds true for TNBC cell lines other than Hs578T. Indeed, doxycycline-induced knockout of *ABCG2* sensitized Hs578T cells to THZ531, THZ1, CDKI-73 and PHA-767491, but barely to SR4835 and not to flavopiridol, thus following similar trends as for combined inhibition with lapatinib (Fig. 3A, Additional file 1: Fig. S6A and B). *ABCG2* knockout also sensitized SKBR7 cells most strongly to THZ531, THZ1 and CDKI-73 (Additional file 1: Fig. S6A and C). In MDA-MB-231 cells, *ABCG2* knockout mostly sensitized to THZ531 and THZ1, but to a lesser extent than in Hs578T, which also is in line with the increased sensitivity to the CDK inhibitors alone and limited sensitization by lapatinib in this cell line, as described above (Additional file 1: Fig. S6D). Moreover, in Hs578T, SKBR7 and MDA-MB-231 cells with ABCG2 knockout, the synergistic interaction of lapatinib with THZ531 was strongly reduced (Additional file 1: Fig. S6E). Similar to genetic knockout, *ABCG2* knockdown also sensitized Hs578T and SKBR7 cells to THZ531, THZ1, CDKI-73 and, to a lesser extent, PHA-767491 (Fig. 3B, Additional file 1: Fig. S7A). Likewise, ABCG2 inhibitor KO143 sensitized Hs578T and SKBR7 cells to THZ531, THZ1, CDKI-73 and PHA-767491 with equal potency as lapatinib (Fig. 3C, Additional file 1: Fig. S7B). Like the combination treatment of various tyrosine kinase inhibitors with THZ531, combination of KO143 with THZ531 arrested cells in the G1/S checkpoint firstly and subsequently induced cell death in Hs578T (Fig. 3D).

Since previous studies have indicated that resistance to THZ1 and THZ531 can also be associated with ABCB1 overexpression [41, 42], we used siRNA knockdown to analyse the importance of other ABC-transporter family members for sensitivity to these compounds. In line with the results of our CRISPR/Cas9 screen, only knockdown of *ABCG2* increased THZ531 sensitivity in Hs578T and SKBR7 cells (Fig. 3E, Additional file 1: Fig. S7D). In addition, unlike KO143, ABCB1 inhibitor verapamil did not sensitize Hs578T or SKBR7 cells to THZ531 (Fig. 3F, Additional file 1: Fig. S7E).

### Kinase inhibitors sensitize TNBC to transcriptional CDK inhibitors by inhibiting ABCG2 activity

**Fig. 4:**
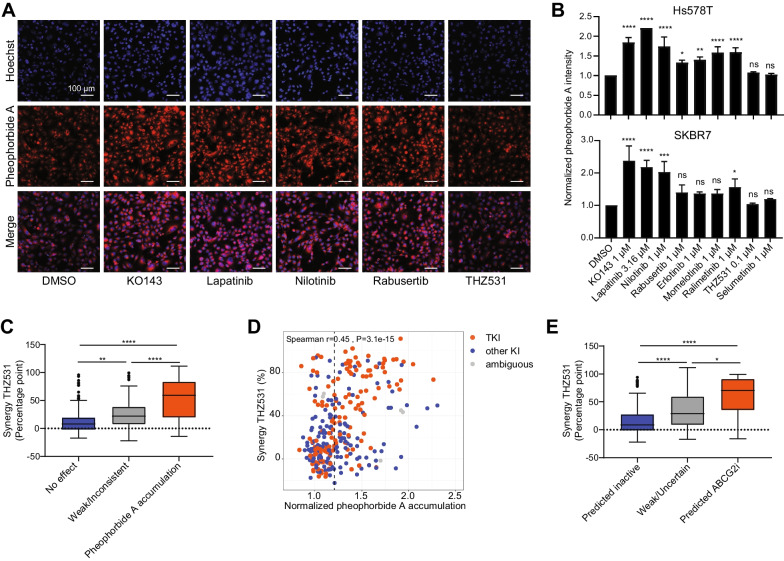
Tyrosine kinase inhibitors target ABCG2 function. **A** Representative images of ABCG2 fluorescent substrate Pheophorbide A upon treatment with KO143 (1 µM), lapatinib (3.16 µM), nilotinib (1 µM), rabusertib (1 µM) and THZ531 (0.1 µM) in Hs578T cells. Scale bar is 100 µm. **B** Relative pheophorbide A intensity upon treatment with inhibitors in Hs578T (upper) or SKBR7 (below) cells. Data are the normalized mean (± SD) of three independent experiments, means were compared using one-way ANOVA with Tukey’s correction for multiple testing. **C** Association of pheophorbide A accumulation with the amount of synergy THZ531 (difference in proliferation (percentage point) in combination treatment compared to additive effects of single treatments) of these kinase inhibitors with THZ531. Kinase inhibitors were screened at 1 µM and were classified (See methods) as having “No effect” (*n* = 112), “Weak/Inconsistent” (*n* = 83) or inducing “Pheophorbide A accumulation” (*n* = 103). Kinase inhibitors that already strongly affected proliferation as individual treatment (proliferation DMSO < 40%) were excluded, and synergy was determined by the difference between individual effects of THZ531 and KI’s. Means were compared using Kruskal–Wallis test with Dunn’s correction. **D** Spearman correlation of amount of synergy of kinase inhibitors with THZ531 (0.1 µM) and the normalized pheophorbide A accumulation upon treatment with these kinase inhibitors alone. **E** Association of predicted ABCG2 inhibitory activity of kinase inhibitors with the amount of synergy of these kinase inhibitors with THZ531. Kinase inhibitors were scored as inactive (*n* = 109), uncertain (*n* = 182), or active (*n* = 31) towards ABCG2 depending on the inhibitory score from the modelling. Kinase inhibitors that already strongly affected proliferation as individual treatment (proliferation DMSO < 40%) were excluded. Means were compared using Kruskal–Wallis test with Dunn’s correction

Although previous studies have also reported that some tyrosine kinase inhibitors, including lapatinib and nilotinib, can inhibit ABCG2 activity, this has not yet been demonstrated for the entire set of kinase inhibitors that synergized with THZ531 [43]. To investigate whether the selected kinase inhibitors in fact inhibit ABCG2 at the synergistic concentrations, we evaluated ABCG2 activity by measuring intracellular accumulation of the fluorescent ABCG2 substrate pheophorbide A [44, 45]. Indeed KO143, lapatinib, nilotinib, momelotinib, ralimetinib and rabusertib caused an increase in intracellular pheophorbide A intensity in Hs578T and SKBR7 cells, suggesting inhibition of ABCG2 activity (Fig. 4A and B). MEK inhibitor selumetinib, that did not synergize with THZ531, and THZ531 itself, did not affect pheophorbide A accumulation.

Next, we examined whether this is a general mechanism of synergy between THZ531 and the identified kinase inhibitors. We therefore screened the kinase inhibitor library for effects on pheophorbide A accumulation. In line with a general mechanism of synergy, we identified 103 kinase inhibitors (from a total of 303 evaluated with less than 40% effect on proliferation at used concentration) that caused pheophorbide A accumulation (fold change > 1.2). These kinase inhibitors also showed a higher extent of synergy in the previously described kinase inhibitor combination screen with THZ531, compared to the 112 kinase inhibitors that did not affect pheophorbide A accumulation (Fig. 4C, Additional file 4: Table S3). Although the extent of pheophorbide A accumulation could not fully describe the variation in extent of synergy with THZ531, there was a significant correlation, showing that a higher pheophorbide A accumulation is associated with increased potentiation of THZ531 treatment (Spearman *r* = 0.45, *P* < 0.0001, Fig. 4D). To explore the relationship between ABCG2 activity and sensitivity to THZ531 further, we used computational modelling to predict the probability of the inhibitory activity of the kinase inhibitors for ABCG2 based on several structural scaffolds described previously [32, 33] (see Methods). This model assesses the interaction profile of small molecules towards ABCG2 using a machine learning approach. It could be found that kinase inhibitors predicted to be inhibitors of ABCG2 synergized more strongly with THZ531 than those predicted to be inactive (Fig. 4E, Additional file 4: Table S3). Indeed, compounds with predicted ABCG2 inhibitory activity also had a higher extent of pheophorbide A accumulation (Additional File 1: Fig. S8). To conclude, these data indicate that a striking number of (mainly tyrosine) kinase inhibitors synergize with THZ531 and other transcriptional CDK inhibitors by inhibiting ABCG2 transport in TNBC cells.

### Combination treatment enhances inhibition of RNA polymerase II by THZ531

**Fig. 5:**
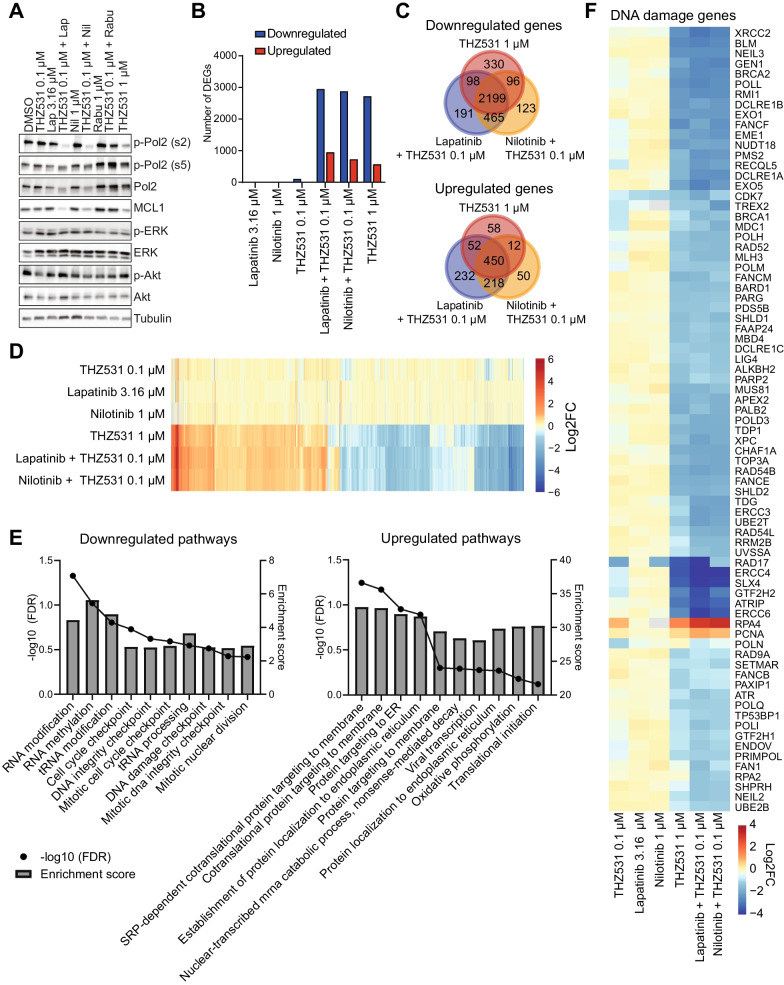
Tyrosine kinase inhibitors sensitize Hs578T cells to transcriptional inhibition by THZ531. **A** Western blot showing protein levels of (phosphorylated) C-terminal domain of RNA polymerase II (Pol2), ERK, Akt, MCL1 and tubulin 24 h after treatment with THZ531 (0.1 µM), lapatinib (3.16 µM), nilotinib (1 µM), rabusertib (1 µM), a combination thereof, or THZ531 (1 µM). Western blot images are representative of three independent experiments. **B** Number of strongly differentially downregulated (Log2 FC <  − 1, Adjusted *p* < 0.05) or upregulated (Log2 FC > 1, Adjusted *p* < 0.05) genes after 8 h of lapatinib (3.16 µM), nilotinib (1 µM), THZ531 (0.1 µM), their combination, or THZ531 (1 µM) treatment as determined by RNA sequencing in Hs578T cells. **C** Overlap of strongly down- and upregulated genes between combination treatments with THZ531 (0.1 µM) and high dose THZ531 (1 µM). **D** Heatmap showing unsupervised clustering of Log2 FCs of the differentially expressed genes (Log2 FC <  − 0.5/> 0.5, adjusted *p* < 0.05) in one of the conditions. **E** Enrichment score and − log10 FDR *p* values of gene ontology pathway enrichment of ranked Log2 FC upon combination treatment with lapatinib (3.16 µM) and THZ531 (0.1 µM). **F** Heatmap showing unsupervised clustering of Log2 FC of strongly differentially expressed (Log2 FC > 1 or <  − 1, adjusted *p* < 0.05) DNA damage genes in one of the conditions. All shown data are from RNA sequencing analysis performed on samples derived in three independent experiments

To further substantiate the role of ABCG2 in the synergistic interactions between THZ531 and other kinase inhibitors, as opposed to other mechanisms such as adaptive responses or targeting of specific pathways, we investigated how the activity and expression of the targets of the synergistic treatments are affected upon treatment. Corresponding to decreased THZ531 efflux by ABCG2 inhibition, the combination treatments with THZ531 (0.1 µM) and lapatinib, nilotinib or rabusertib synergistically reduced phosphorylation of RNA polymerase II (Ser2) and to a lesser extent total expression of RNA polymerase II, in a similar fashion as THZ531 at a higher dose (1 µM), in Hs578T and SKBR7 cells (Fig. 5A, Additional file 1: Fig. S9A and B). Moreover, the short-lived pro-survival protein MCL-1, which is often downregulated by transcriptional CDK inhibitors as a direct result of transcriptional inhibition [46], was also downregulated upon treatments with combinations of THZ531 with lapatinib or nilotinib, or a high dose of THZ531.

In accordance with ABCG2 inhibition as the main mechanism of synergy, and not adaptive signalling or simultaneous inhibition of a specific pathway, we did not observe clear changes within MAPK or PI3K pathways, the key downstream pathways of EGFR signalling, with the individual or combination treatments after 24 h in Hs578T or SKBR7 cells (Fig. 5A and Additional file 1: Fig. S9A). Although after 48 h the combination treatment of lapatinib and THZ531 slightly increased levels of p-MEK and p-ERK, this may be related to induction of apoptosis, rather than stimulation of these growth factor pathways (Additional file 1: Fig. S9B). Indeed, the combination of lapatinib and THZ531 caused PARP cleavage at 48 h in both Hs578T and SKBR7 cells, indicative of caspase-dependent apoptosis. Collectively, these data show that the tyrosine kinase inhibitors lapatinib and nilotinib mainly sensitize the cells towards the inhibitory effect of THZ531 on RNA polymerase II activity, which subsequently leads to apoptotic cell death.

### Tyrosine kinase inhibitors sensitize cells to transcriptional repression by THZ531

To explore whether additional mechanisms contribute to the effect of ABCG2 inhibition on THZ531 response, we studied the transcriptional changes caused by THZ531 (0.1 or 1.0 μM), lapatinib (3.16 μM), nilotinib (1.0 μM) and combination treatments. RNA sequencing on samples collected 8 h after treatment revealed that mainly combination treatments of 0.1 μM THZ531 with lapatinib or nilotinib and treatment with 1.0 μM THZ531 alone affected transcription, predominantly resulting in downregulation of gene expression (2953, 2883 and 2723 genes, respectively, log2 fold change (FC) < − 1, adjusted *p* < 0.05; Fig. 5B; Additional file 5: Table S4). Corresponding to the increased sensitivity to THZ531 caused by lapatinib or nilotinib mediated ABCG2 inhibition, the differences in gene expression caused by the treatment with a high dosage of THZ531 alone were nearly identical to the effects by the combination treatments (Fig. 5C and D). This further supports that nilotinib and lapatinib are sensitizing the tumour cells to THZ531 and further rules out that the synergy is occurring because THZ531 is suppressing adaptive transcriptional responses. Only a limited number of genes got slightly upregulated upon treatment with lapatinib alone (56 genes, log2 FC > 0.5, adjusted *p* < 0.05) or nilotinib alone (72 genes, log2 FC > 0.5, adjusted *p* < 0.05) (Additional file 1: Fig. S10A and B).

GO biological processes, that were enriched among the genes downregulated by combination treatment with THZ531 and lapatinib, were mostly related to regulation of RNA expression, cell cycle progression and DNA damage checkpoints (Fig. 5E). Biological processes, which were enriched among the upregulated genes, included mainly processes involved in translation and protein localization. Previous studies have highlighted that CDK12/13 inhibitors mainly disrupt the expression of DNA damage genes [5–8]. Although the effects in our study are not limited to these genes, we do also observe that DNA damage response genes are mostly downregulated upon combination treatment with THZ531 (0.1 µM) or high dosage of THZ531 (1 µM) (Fig. 5F, Additional file 1: Fig. S10C). Moreover, compared to other genes, these treatments more specifically reduced the expression of many transcription factors, including mostly zinc finger (ZNF) transcription factors (Additional file 1: Fig. S10C and D). Altogether these data show that CDK12/13 inhibition with THZ531 strongly disrupts gene expression and further supports that combination with lapatinib or nilotinib sensitizes cells to THZ531 to inhibit CDK12/13-regulated gene expression.

### Tyrosine kinase inhibitors sensitize cells to CDK12/13-related intronic polyadenylation defects caused by THZ531

**Fig. 6:**
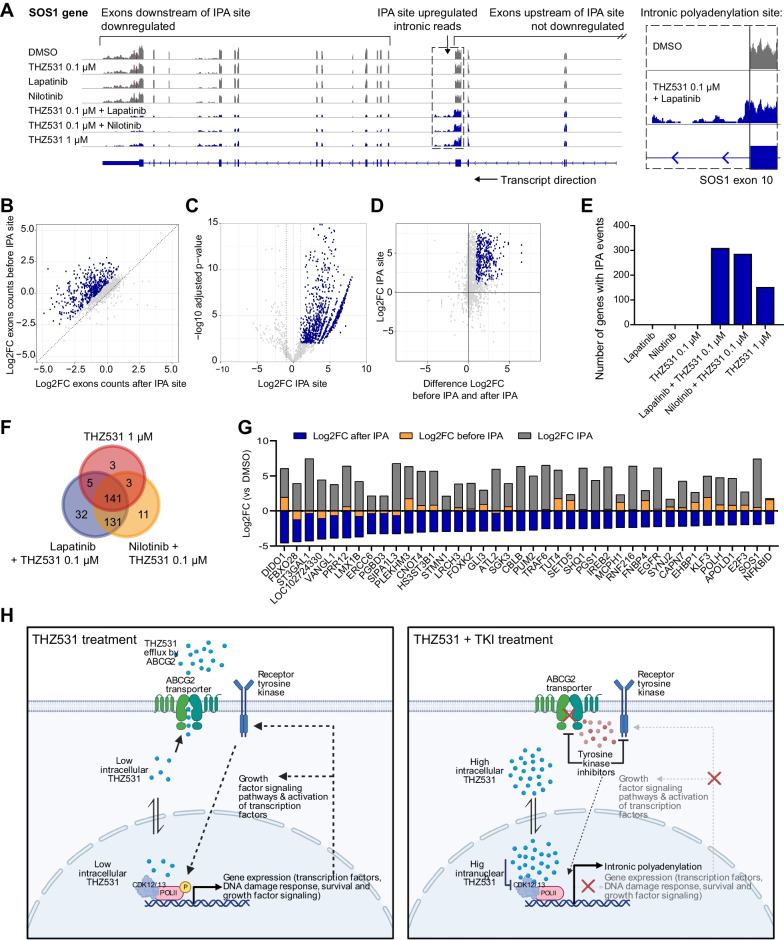
Tyrosine kinase inhibitors sensitize Hs578T cells to intronic polyadenylation induction by THZ531. **A** Gene track example of the SOS1 gene from RNA sequencing data upon 8 h treatment with THZ531 (0.1 µM), lapatinib (3.16 µM), nilotinib (1 µM), a combination thereof, and high dose THZ531 (1 µM), showing intronic polyadenylation (IPA) for the combination treatments and high dose THZ531. **B** Quantification of differential exon expression (Log2 FC) downstream and upstream of the IPA site, blue dots indicate genes with significantly downregulated exons downstream of (after) the IPA site compared to upstream of (before) the IPA site (difference Log2 FC before and after > 0.75, adjusted *p* values of exons after IPA < 0.01). **C** Quantification of differential expression (Log2 FC and Adjusted *p* value) of the IPA site itself, blue dots indicate significantly increased expression of IPA sites (adjusted *p* < 0.001, Log2 FC > 1). **D** Presentation of significant IPA events (blue) showing events that had a significantly decreased exon expression downstream of the IPA site and had significantly increased expression of the IPA site itself. **E** Number of genes with significant IPA events upon treatment with the different conditions. **F** Overlap of genes with significant IPA events in the combination treatments (Lapatinib/Nilotinib + THZ531 0.1 µM) versus high dose THZ531 (1 µM). **G** Log2 FC of exons upstream and downstream of the IPA site and of the IPA site of the top 40 genes with most strong IPA event after treatment with lapatinib and THZ531 (0.1 µM), sorted on lowest Log2 FC of exons after IPA site. **H** Illustration of mechanism of synergy between THZ531 and kinase inhibitors via ABCG2 transporter inhibition. Illustration was created with BioRender.com

Previous studies have demonstrated that a key mechanism behind transcription regulation by CDK12/13 inhibitors is an increase in premature cleavage and polyadenylation in introns, called “intronic polyadenylation” (IPA) [6, 8]. Indeed, our polyA-enriched RNA sequencing data also showed gene track patterns of upregulated intronic polyadenylation with concomitant downregulation of transcription of downstream exons after treatment with THZ531, for example in the *SOS1* and *JAK2* genes (Fig. 6A, Additional file 1: Fig. S11A). The combination treatment of lapatinib and nilotinib with THZ531 (0.1 µM) or a high dose of THZ531 (1 µM) more strongly reduced the expression of exons after (downstream of) potential IPA sites compared to the expression of exons before (upstream of) these sites, suggesting a premature end of the transcript around these IPA sites (Fig. 6B, Additional file 1: Fig. S11B). Moreover, these treatments increased the expression of many of these potential IPA sites themselves (Fig. 6C). Taken together, while the individual treatments with lapatinib, nilotinib and low concentration of THZ531 (0.1 µM) did not affect intronic polyadenylation, lapatinib and nilotinib sensitized the cells to CDK12/13-related defects in transcriptional processing by THZ531 (Fig. 6D–F and Additional file 6: Table S5).

Previous studies have demonstrated that IPA upon CDK12/13 inhibition mostly occurs in DNA damage response genes due to their relatively long length [6, 8]. Indeed, the high dose of THZ531 and combination treatments cause IPA of several genes, which are enriched for genes involved in DNA damage response pathways, such as *ERCC6* (Fig. 6G, Additional file 1: Fig. S12A and B). However, our data demonstrate that also genes from other pathways are affected, including genes involved in processes such as regulation of transcription (e.g. *SMARCA4*), cell cycle (e.g. *E2F3*) and protein tyrosine kinase signalling (e.g. *EGFR*) (Fig. 6G and Additional file 1: Fig. S12A, B). Interestingly, several of these genes are associated with pathways targeted by lapatinib or nilotinib, including *EGFR*, *JAK2, BRAF*, and *SOS1*. Although IPA of these genes is likely not the driving force behind the synergy, this disruption of gene processing could further hamper the growth of TNBC cells with the addition of these treatments. Overall, these data further support that tyrosine kinase inhibitors sensitize cells to THZ531 and thereby CDK12/13 related transcriptional defects by inhibiting ABCG2-mediated drug efflux (Fig. 6H).

## Discussion

Despite tremendous efforts to establish new effective targeted therapies, TNBC patients still have a very poor clinical prognosis. This study focused on the systematic exploration of potential combination treatments with transcriptional CDK inhibitors in the context of TNBC. Using unbiased, high-throughput approaches, including kinase inhibitor (combination) screening, genome-wide CRISPR knockout sensitization screening and cell line biomarker profiling, we identified a large amount of kinase inhibitors that synergize with CDK12/13 inhibitor THZ531 and demonstrated that this predominantly occurs via inhibition of ABCG2 (illustrated in Fig. 6H).

Upregulation of ABC transporters ABCB1 and ABCG2 has previously been described as a mechanism of acquired resistance to the structurally similar CDK7 inhibitor THZ1 [41, 42] and causes cross-resistance to THZ531 [41]. Our data show that ABCG2 is the key determinant of THZ531 sensitivity in CDK-inhibitor treatment-naïve TNBC cell lines. In addition, we show that ABCG2 transporter activity also decreases the response to CDK7 inhibitor THZ1, CDC7/CDK9 inhibitor PHA-767491 and CDK9 inhibitor CDKI-73. Furthermore, this study systematically demonstrated the importance of ABCG2 for the synergistic interactions between THZ531 and other kinase inhibitors. Although the interaction of ABCG2 with several tyrosine kinase inhibitors, including lapatinib and nilotinib, has been previously described [43], our study points out a wide range of kinase inhibitors as ABCG2 inhibitors. Of note, ABCG2 inhibitors are frequently also ABCG2 substrates that occupy the transporter competitively [47, 48], and the extent of ABCG2 inhibition may be context-dependent (e.g. depending on transporter affinity of both compounds, expression of ABCG2 and efflux by other drug transporters) and remains to be further investigated. Future studies should also investigate whether or not these kinase inhibitors could be a better strategy for overcoming ABCG2-mediated drug resistance than selective ABCG2 inhibitors in terms of additional acquired drug resistance and safety. For example, some of these kinase inhibitors may be more effective by simultaneously inhibiting other ABC transporters (e.g. ABCB1), or by simultaneously inhibiting TNBC drivers (e.g. EGFR), yet at the same time may also induce more side-effects. Nevertheless, these findings underline the general importance of evaluating the role of ABC transporters in both drug resistance and drug synergism.

The effects of combination treatment with THZ531 and lapatinib or nilotinib on transcription are strikingly similar to treatment with a higher concentration of THZ531 alone, confirming that synergistic kinase inhibitors mainly increase the sensitivity of the tumour cells to THZ531. In line with other studies, we observed downregulation of DNA damage response genes and disruption of gene processing, causing intronic polyadenylation [5–8]. Although these previous studies described that CDK12/13 inhibition predominantly induced intronic polyadenylation within DNA damage gene transcripts, our study demonstrates that CDK12/13 inhibition also induces intronic polyadenylation in transcripts from genes contributing to growth factor signalling, transcription and the cell cycle.

Synergy between transcriptional CDK inhibitors and other inhibitors has been widely, yet separately, described in previous studies [11, 13, 16, 18–21, 49]. Some of these studies have already previously shown that synergistic combinations of lapatinib and erlotinib with transcriptional CDK inhibitor THZ1 is effective and safe in vivo [13, 49]. Here, we confirmed many of the previously described synergistic interactions with kinase inhibitors, and identified much more new synergistic combinations, in the context of TNBC. Importantly, the findings described in the current study could also provide an (alternative or additional) mechanism to the previously reported synergistic interactions of transcriptional CDK inhibitors with kinase inhibitors. These studies frequently did not focus on the mechanism behind these interactions, and/or proposed different mechanisms of synergy, namely that THZ1 or THZ531 can prevent adaptive signalling [11, 13, 16] or interfere with the same pathway as the combined drug [18–21]. Yet, these data often do not rule out ABCG2, or other ABC transporters (e.g. ABCB1), from being involved in this interaction. Evidence showing that CDK7/CDK12/CDK13 depletion, which would not be affected by ABCG2 interactions, elicits similar synergy as inhibitor treatment is mostly limited in these studies. Moreover, observations of THZ1/THZ531 disrupting a specific pathway, also affected by the synergistic combination agent, are often only evident at higher concentrations of the inhibitors, or in the combination treatment, but not at the individual lower doses used in the combination treatment. This suggests that these effects may be a consequence of the synergy, rather than a cause of it. Interestingly, while synergistic interactions are previously described between THZ1 or THZ531 and multiple kinase inhibitors (e.g. lapatinib, erlotinib, ralimetinib, ponatinib and nilotinib) [11, 13, 18, 21, 49], we observe that these kinase inhibitors can also inhibit ABCG2 activity. The role of ABCG2, in these, and perhaps other, synergistic interactions, should be further determined in these specific models to understand which synergistic interactions are mediated through ABCG2, or other ABC transporters, and which are not.

Overcoming ABCG2-mediated drug resistance using tyrosine kinase inhibitors may thus ultimately benefit the anti-cancer efficacy of transcriptional CDK inhibitors and other ABCG2 substrates in the clinic. Many efforts from clinical trials combining mostly ABCB1 inhibitors, but also ABCG2 inhibitors, with multiple chemotherapeutic agents have failed due to either limited efficacy or dose-limiting toxicity. However, these studies have often not selected patients based on tumour ABCG2 expression and have been mostly performed with cytostatic agents with a narrow therapeutic window [50]. In contrast to ABCB1, ABCG2 protein and mRNA expression has been demonstrated in breast cancer, and can be linked to therapy response [51–53]. Importantly, ABCB1/ABCG2 inhibitor dofequidar improved therapy responses in a subset of breast cancer patients [54]. In addition, inhibition of ABCG2 efflux in other tissues might improve drug pharmacokinetics, which could be especially relevant for orally administered drugs or drugs that need to cross the blood–brain barrier, for example to treat glioblastomas. Although a clinical strategy for safe and synergistic ABCG2 inhibition with kinase inhibitors remains to be established, this study contributes to the identification of drugs that could be used to inhibit ABCG2 and CDK inhibitors that are transported by ABCG2.

## Conclusions

This study demonstrates that many tyrosine kinase inhibitors can sensitize TNBC cells to transcriptional CDK inhibition, especially THZ531, by ABCG2 inhibition. This study urges for a paradigm shift where the potential role of ABC transporters in synergistic drug–drug interactions, especially with transcriptional CDK inhibitors, should be critically evaluated. Ultimately, this strategy could improve clinical efficacy of ABCG2 substrates, in particular transcriptional CDK inhibitors, and aid in developing new strategies for the treatment of TNBC.

## Supplementary Information

**Additional file 1: Fig. S1**. Combination treatment of lapatinib with CDK inhibitors. A Proliferative responses upon treatment with dose ranges of lapatinib in combination with pan-CDK inhibitor flavopiridolin Hs578T cells. Data are the meanof three independent experiments. B, C Proliferative responses upon treatment with dose ranges of lapatinib in combination with CDK9/CDC7 inhibitor PHA-767491, CDK9 inhibitor CDKI-73, CDK9 inhibitor BAY-1143572, CDK7/12/13 inhibitor THZ1, CDK12/13 inhibitor THZ531, pan-CDK inhibitor flavopiridoland CDK12/13 inhibitor SR4835in MDA-MB-231or SKBR7cells. Data are the meanof three independent experiments. D Corresponding individual bliss synergy scores and meanfor the combination treatment at each concentration.** Fig. S2**. Kinase inhibitor combination screening with THZ1 and THZ531. A Proliferative responses in kinase inhibitorscreening upon treatment with KI’s onlyor in combination with THZ531 in Hs578Tand MDA-MB-231. Colours indicate the pathways as classified in the Selleckchem library. B Proliferative responses in KI screening upon treatment with KI onlyor in combination with THZ1 in Hs578Tand MDA-MB-231. Data are the meanof two independent experiments. C Tukey’s boxplots showing percentage point of synergy between THZ531 and tyrosine KIor other KI. Means were compared using Mann–Whitney test. D, E Proliferation upon treatment with dose ranges of nilotinib, rabusertib, erlotinib, momelotinib, ralimetinib alone or in combination with THZ531in SKBR7, or with THZ531in MDA-MB-231. Data are the meanof fouror threeindependent experiments.** Fig. S3**. FUCCI and cell death dynamics upon treatment with THZ531 and kinase inhibitors. A FUCCI cel cell cycle progression in Hs578T cells 12, 18, 24, 48 and 72 hours after treatment with lapatinib, nilotinib, rabusertib, erlotinib, momelotinib and ralimetinibalone or in combination with THZ531and single treatment of high dose THZ531or cisplatin. Cdt1-positive cells are in G1 phase, Cdt1- and Geminin-positive cells are stalled at the G1/S checkpoint, Geminin-positive cells are in S, G2 or M-phase and negative cells are in transition from M to G1 phase. B, C Induction ofcell death measured by Annexin V and PI staining in Hs578Tor SKBR7after 12, 18, 24, 48 and 72 hours of treatment with these inhibitors. Cell death and FUCCI data are the meanof three independent experiments.** Fig. S4**. Dose responses of combination treatment of THZ531 with lapatinib and nilotinib in TNBC cell line panel. Proliferative responses of TNBC cell lines and ER+ BC MCF7 and T47D cell lines to single and combination treatment of lapatinib and nilotinib with THZ531 0.0316 and 0.1 µM. Data were used to calculate BLISS synergy scores. Cell lines coloured in blue were synergistically affected by these combination treatments, while cell lines in red were not. For cell lines indicated in grey only weak synergy or synergy for only one of the compounds was observed. Data are the meanfrom three independent replicates. For MDA-MB-468, MDA-MB-436 and SUM159PT data are meanfrom two independent replicates.** Fig. S5**. Pharmacological interaction and ABCG2 are key to synergistic interaction of lapatinib and nilotinib with CDK12/13 inhibitor THZ531. A CRISPR-Cas9 dropout screening results showing the Log2 FC of THZ531 versus DMSO of gene knockouts and their effects on proliferation without treatment. Hits that were significantly dropped out upon treatment with THZ531 are highlighted. B Rank-ordered RNA sequencing-based mRNA expression from gene set enrichment analysis of THZ531-resistant and synergistic cell lines versus THZ531-sensitive and non-synergistic cell lines showing the top and bottom 10 genes correlated with this phenotype. C Lack in overlap between targets of synergistic kinase inhibitors and potential interactors of themwith the top 20 gene associations of the resistance/sensitive phenotype from RNA-sequencing or RNA microarray data, and with significant hits from CRISPR-Cas9 knockout sensitization screening with THZ531. D Proliferative responses of Hs578Tand MDA-MB-231cells with dox-inducible Cas9 and ABCG2 gRNA to treatment with dose range of lapatinib or nilotinib, with and without doxycycline. E Western blots showing reduced CDK12 protein expression in ABCG2 gRNA expressing Hs578T or MDA-MB-231 cells after addition of doxycycline. F Proliferative responses to treatment with lapatinibor nilotinibin Hs578T cells with CDK12, CDK13 or CDK12/CDK13knockdown. Data are the meanof three independent experiments or representativeof three independent experiments.** Fig. S6**. ABCG2 knockout synergizes with THZ531 in similar fashion as synergistic kinase inhibitors. A Knockout efficiency of ABCG2 4 days after doxycycline induction in Hs578T, SKBR7 and MDA-MB-231 iCas9 cells expressing ABCG2 gRNA, as determined by %indel using TIDE compared to controls without doxycycline induction. Data are meanof two independent experiments. B Proliferative response to transcriptional CDK inhibitors for Hs578T cells with non-targeting gRNA ctrl with and without doxycyclinetreatment, showing no effect of doxycycline induction itself. C, D Proliferative response to transcriptional CDK inhibitors for SKBR7or MDA-MB-231cells expressing ABCG2 gRNA after treatment without doxycyclineor with doxycycline. E Effect of combination treatment with lapatiniband THZ531 in Hs578t, SKBR7 and MDA-MB-231 in controlversus ABCG2 knockout. All proliferation data are the meanfrom three independent experiments.** Fig. S7**. ABCG2 inhibition and knockdown synergizes with THZ531 in similar fashion as synergistic kinase inhibitors in SKBR7 cells. A Proliferative response to transcriptional CDK inhibitors in SKBR7 cells treated with transfection reagent, siKPor siABCG2. B Proliferative responses of dose ranges of transcriptional CDK inhibitors alone, or in combination with KO143or lapatinibin SKBR7 cells. C Effects of combination treatment of KO143with THZ531on AnnexinV/PI stained cell deathin SKBR7 cells. D ABC-transporter siRNA screen showing proliferative responses upon knockdown of ABC-transporter genes together with DMSO or THZ531treatment in SKBR7 cells. E Proliferative responses of combination treatment of dose range of either ABCG2 inhibitor KO143 or ABCB1/ABCC1 inhibitor Verapamil with THZ531 0.1 µM in SKBR7 cells. All knockdown data are the meanfrom two independent experiments. All data from combination treatments are the meanfrom three independent experiments.** Fig. S8**. Pheophorbide A accumulation is higher for compounds predicted to have ABCG2 inhibitory activity. Bar graphs represent the median normalized pheophorbide A accumulation, with 95% confidence intervals. Mean ranks were compared using Kruskal–Wallis testing and corrected for multiple testing using the Benjamini and Hochberg method.** Fig. S9**. Protein levels after treatment with kinase inhibitors and THZ531. A Western blot showing protein levels ofC-terminal domain of RNA polymerase II, ERK, MEK, MCL1, total and cleaved PARP and tubulin 24 hours after treatment with THZ531, lapatinib, nilotinib, rabusertib, a combination thereof, or THZ531in SKBR7 cells. P-Akt levels were not detectable in SKBR7 cells. B Western blot showing the levels ofC-terminal domain of RNA polymerase II, ERK, Akt, MEK, MCL1, total and cleaved PARP and tubulin after 0, 1, 2, 4, 6, 24 and 48 hours treatment with the combination treatment of lapatiniband THZ531in Hs578T and SKBR7 cells. Western blot images are representative of two independent experiments.** Fig. S10**. Differentially expressed genes in Hs578T upon combination treatment with THZ531. A, B Log2 FCs of genes significantly up- or downregulatedupon treatment with single nilotinibor lapatinibtreatment. nilotinib or lapatinib upregulated genes that are not as strongly upregulated in the combination treatment are highlighted by the vertical line. C Cumulative fraction plots showing gene expression changes of all genes versustranscription factors or DNA damage genes. Distributions were compared using Kolmogorov-Smirnov tests. D Selection of transcription factor genes strongly up- or downregulatedin at least one of the treatments.** Fig. S11**. Analysis of intronic polyadenylation in RNA sequencing data. A Example of gene tracks for the JAK2 gene, showing intronic polyadenylationfor all the independent replicates treated with lapatinib/Nilotinib and THZ531 0.1 µM or high dose THZ531 1 µM. B Schematic of IPA and its effect on polyA-enriched sequencing data and sequencing tracks. Possible intronic polyadenylation sites, with polyadenylation signals, are described in the polyAsite atlas and the region of −10 and +10 nucleotides was counted using HTseq-count. Differential expression of these IPA sites was analyzed using Deseq2. Individual exons were counted using DexSeq-count and the differential expression of the sum from all exons upstreamof the IPA site and downstreamof the IPA site was determined using Deseq2. Intronic polyadenylation events were considered significant when both the IPA site itself was significantly upregulatedand the difference between the expression of exons before and after the IPA site was larger than 0.75, while the exons after the IPA site were significantly downregulated.** Fig. S12**. Pathways affected by IPA after treatment with lapatinib and THZ531. A Top 30 significantly enriched pathwaysamong genes with IPA event upon treatment with lapatinib and THZ531. B Gene network showing interactions between genes with IPA event and protein-protein interactionenrichment p value. Disconnected nodeswere not shown in the network. Data are from RNA-sequencing analysis performed on samples derived in three independent experiments.** Fig. S13**. Uncropped western blot images. Composite imagesof chemiluminescent or fluorescentsignal with colorimetric signal of the protein ladder, if available. Source imagesof chemiluminescent signal or fluorescent signalused for the cropped images in Fig. 5A, S5E, S9A and S9B. Blots were mostly cut into two pieces to stain for small and large proteins. The approximate cropping areas used for the final images are indicated by the blue squares. Lanes not used for the final image represent irrelevant conditions.

Additional file 2: Table S1. THZ1 and THZ531 kinase inhibitor combination screening proliferation results in Hs578T and MDA-MB-231 cells.

Additional file 3: Table S2. CRISPR-Cas9 knockout sensitization screening results with THZ531. Log2 fold changes and FDR p values of targets of the synergistic kinases, and their main interactors, are highlighted.

Additional file 4: Table S3. Associations of extent of synergy with THZ531 and molecular weight, pheophorbide A accumulation and ABCG2 activity model predictions. Only kinase inhibitors with >40% remaining proliferation upon individual kinase inhibitor treatment are shown. NA values for pheophorbide A results include kinase inhibitors not available in updated libraryor outliers. Kinase inhibitors with NA values for ABCG2 activity predictions were not evaluated

Additional file 5: Table S4. Differential gene expression data from RNA-sequencing upon 8 hours treatment with THZ531, lapatinib, nilotinib and combinations in Hs578T

Additional file 6: Table S5. Quantifications of intronic polyadenylation related counts from RNA-sequencing upon treatment with THZ531, lapatinib, nilotinib and combinations in Hs578T.

## Data Availability

Raw RNA sequencing datasets generated and analysed during the current study are available in the GEO repository (GSE210987). Uncropped western blot images are available in Additional File 1: Fig. S13. Kinase inhibitor screening data and processed RNA sequencing data are available in Additional Files 2–6. All other data and cell lines generated in this study are available from the corresponding author upon reasonable request.

## Abbreviations

CDK: Cyclin-dependent kinases
ER: Oestrogen receptor
FUCCI: Fluorescent cell cycle indicators
GSEA: Gene set enrichment analysis
HER2: Human epidermal growth factor receptor
IPA: Intronic polyadenylation
KI: Kinase inhibitor
PI: Propidium iodide
PR: Progesteron receptor
pp: Percentage point
PVDF: Polyvinylidene fluoride
siKP: SiRNA for non-specific kinase pool
siRNA: Small interfering RNA
SRB: Sulforhodamine B
TCA: Trichloroacetic acid
TKI: Tyrosine kinase inhibitor
TNBC: Triple-negative breast cancer
