# Supplementary material for: Systematic screening identifies ABCG2 as critical factor underlying synergy of kinase inhibitors with transcriptional CDK inhibitors
Source: Breast Cancer Res. 2023 May 5;25:51. doi: 10.1186/s13058-023-01648-x (PMC10161439; doi:10.1186/s13058-023-01648-x)
Supplement: Supplementary file 1 — Additional file 1: Fig. S1. Combination treatment of lapatinib with CDK inhibitors. A Proliferative responses upon treatment with dose ranges of lapatinib in combination with pan-CDK inhibitor flavopiridolin Hs578T cells. Data are the meanof three independent experiments. B, C Proliferative responses upon treatment with dose ranges of lapatinib in combination with CDK9/CDC7 inhibitor PHA-767491, CDK9 inhibitor CDKI-73, CDK9 inhibitor BAY-1143572, CDK7/12/13 inhibitor THZ1, CDK12/13 inhibitor THZ531, pan-CDK inhibitor flavopiridoland CDK12/13 inhibitor SR4835in MDA-MB-231or SKBR7cells. Data are the meanof three independent experiments. D Corresponding individual bliss synergy scores and meanfor the combination treatment at each concentration. Fig. S2. Kinase inhibitor combination screening with THZ1 and THZ531. A Proliferative responses in kinase inhibitorscreening upon treatment with KI’s onlyor in combination with THZ531 in Hs578Tand MDA-MB-231. Colours indicate the pathways as classified in the Selleckchem library. B Proliferative responses in KI screening upon treatment with KI onlyor in combination with THZ1 in Hs578Tand MDA-MB-231. Data are the meanof two independent experiments. C Tukey’s boxplots showing percentage point of synergy between THZ531 and tyrosine KIor other KI. Means were compared using Mann–Whitney test. D, E Proliferation upon treatment with dose ranges of nilotinib, rabusertib, erlotinib, momelotinib, ralimetinib alone or in combination with THZ531in SKBR7, or with THZ531in MDA-MB-231. Data are the meanof fouror threeindependent experiments. Fig. S3. FUCCI and cell death dynamics upon treatment with THZ531 and kinase inhibitors. A FUCCI cel cell cycle progression in Hs578T cells 12, 18, 24, 48 and 72 hours after treatment with lapatinib, nilotinib, rabusertib, erlotinib, momelotinib and ralimetinibalone or in combination with THZ531and single treatment of high dose THZ531or cisplatin. Cdt1-positive cells are in G1 phase, Cdt1- and Geminin-posi [file 13058_2023_1648_MOESM1_ESM.pdf]

Additional File 1: Supplementary Figures

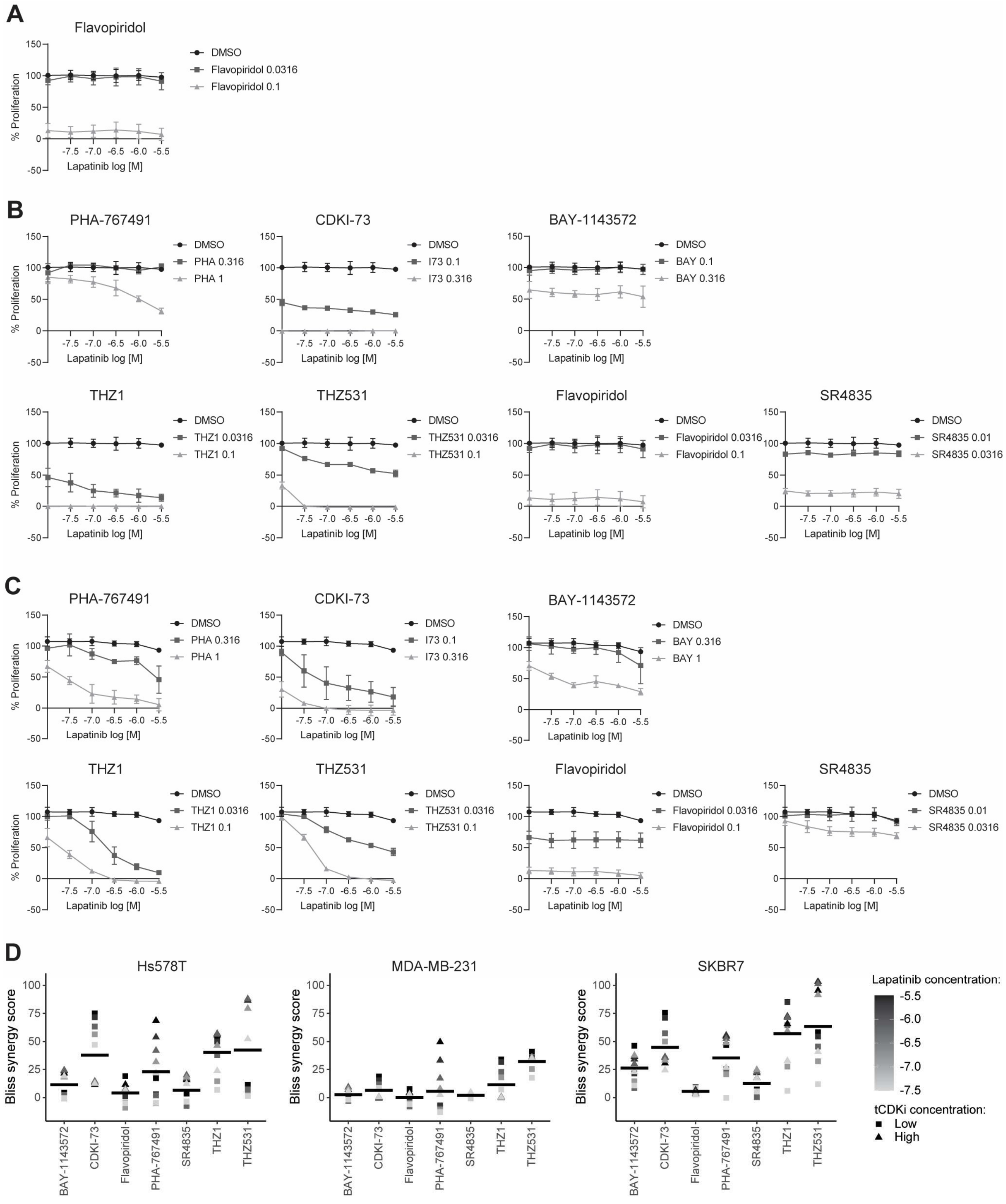

Fig. S1. Combination treatment of lapatinib with CDK inhibitors. **A** Proliferative responses upon treatment with dose ranges of lapatinib in combination with pan-CDK inhibitor flavopiridol (0.0316 and 0.1 µM) in Hs578T cells. Data are the mean (±SD) of three independent experiments. **B,C** Proliferative responses upon treatment with dose ranges of lapatinib in combination with CDK9/CDC7 inhibitor PHA-767491 (PHA 0.316 and 1 µM), CDK9 inhibitor CDKI-73 (0.1 and 0.316 µM), CDK9 inhibitor BAY-1143572 (BAY 0.1 and 0.316 µM), CDK7/12/13 inhibitor THZ1 (0.0316 and 0.1 µM), CDK12/13 inhibitor THZ531 (0.0316 and 0.1 µM), pan-CDK inhibitor flavopiridol (0.0316 and 0.1 µM) and CDK12/13 inhibitor SR4835 (0.01 and 0.0316 µM) in MDA-MB-231 **(B)** or SKBR7 **(C)** cells. Data are the mean (±SD) of three independent experiments. **D** Corresponding individual bliss synergy scores and mean (indicated by thick line) for the combination treatment at each concentration.

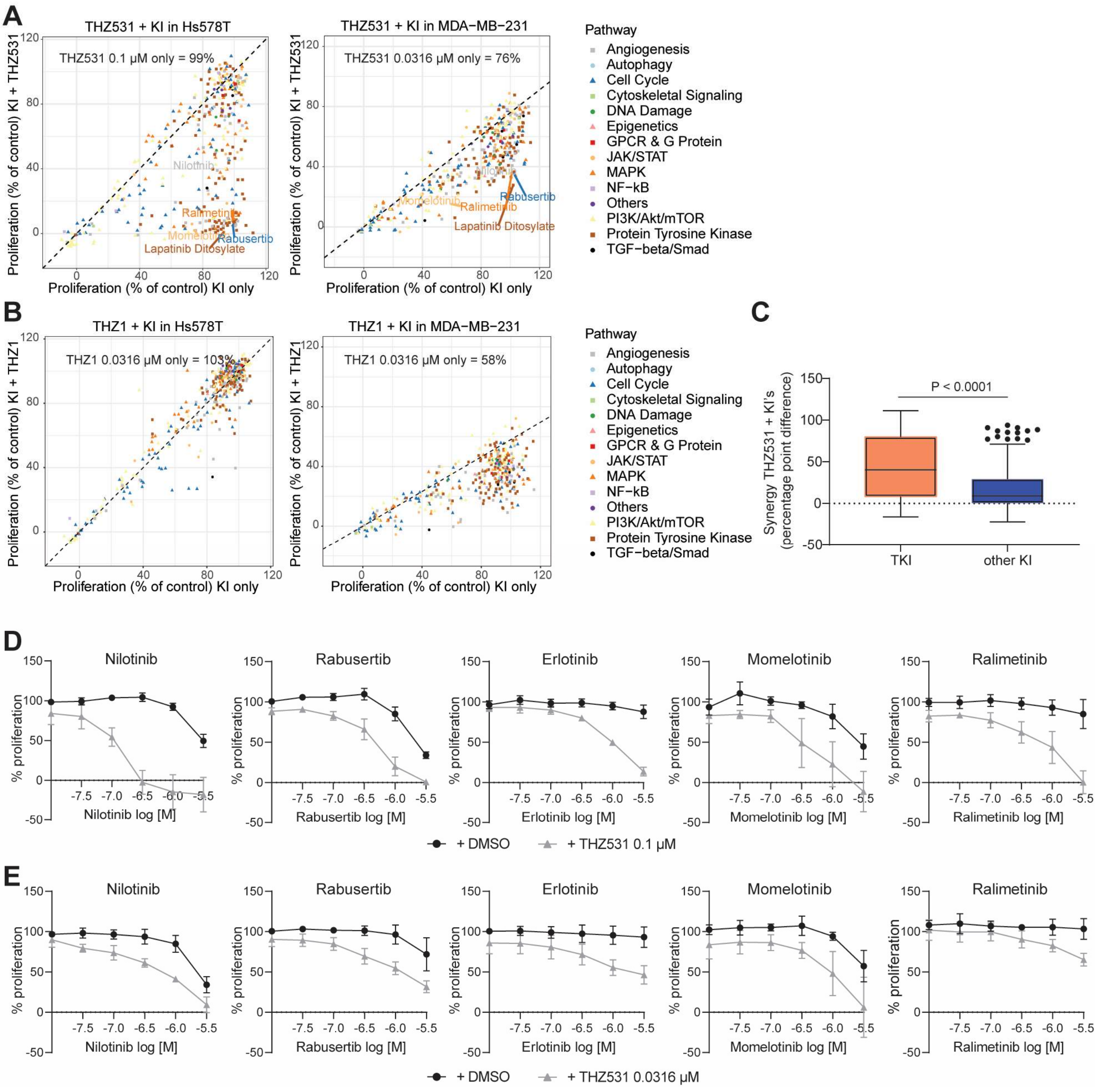

Fig. S2. Kinase inhibitor combination screening with THZ1 and THZ531. **A** Proliferative responses in kinase inhibitor (KI) screening upon treatment with KI's only (1 μM) or in combination with THZ531 in Hs578T (0.1 μM, left) and MDA-MB-231 (0.0316 μM, right). Colours indicate the pathways as classified in the Selleckchem library. **B** Proliferative responses in KI screening upon treatment with KI only (1 μM) or in combination with THZ1 in Hs578T (0.0316 μM, left) and MDA-MB-231 (0.0316 μM, right). Data are the mean (±SD) of two independent experiments. **C** Tukey's boxplots showing percentage point of synergy between THZ531 and tyrosine KI (TKI) or other KI. Means were compared using Mann-Whitney test. **D, E** Proliferation upon treatment with dose ranges of nilotinib, rabusertib, erlotinib, momelotinib, ralimetinib alone or in combination with THZ531 (0.1 μM) in SKBR7 **(D)**, or with THZ531 (0.0316 μM) in MDA-MB-231 **(E)**. Data are the mean (±SD) of four (SKBR7) or three (MDA-MB-231) independent experiments.

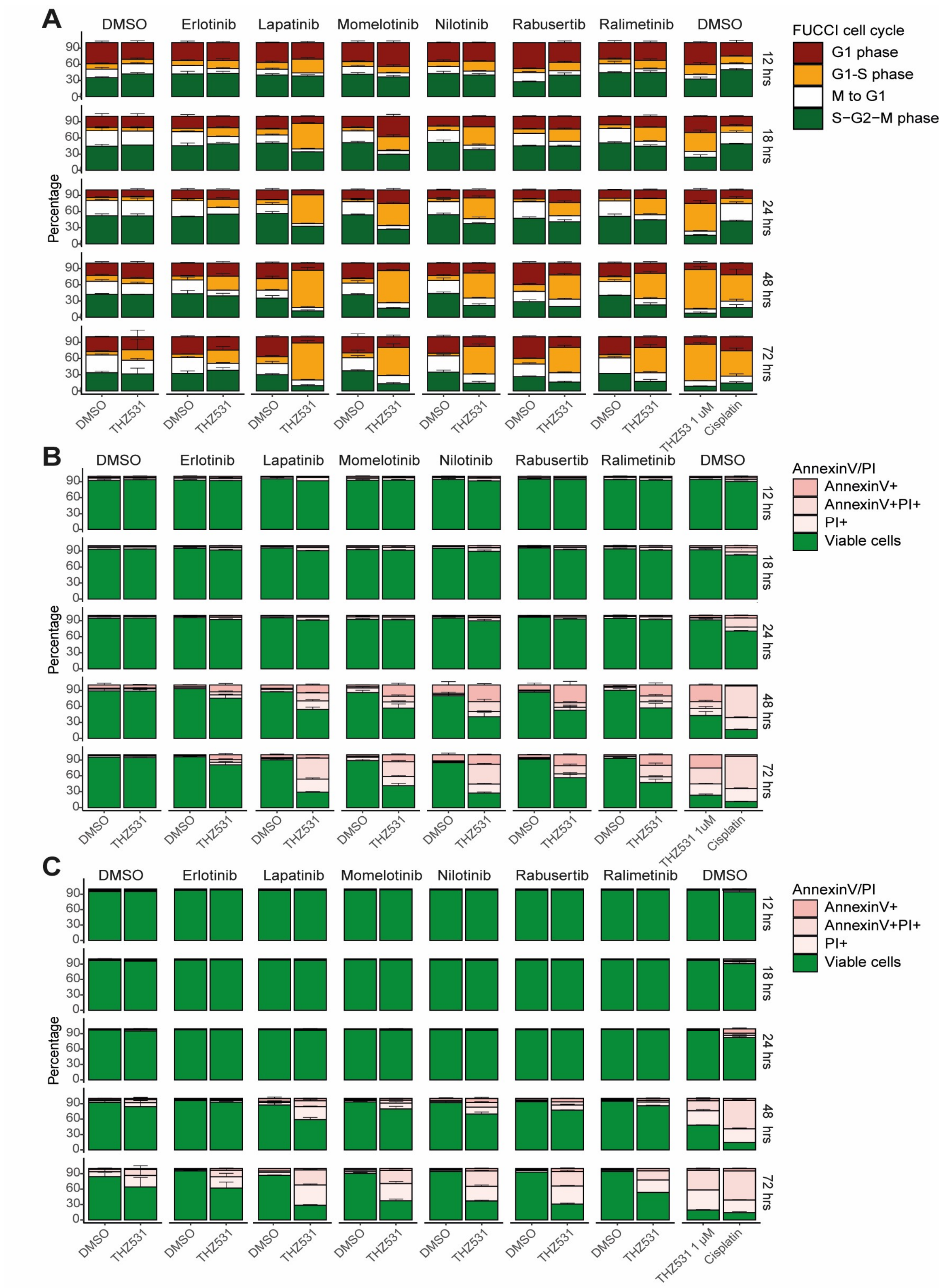

Fig. S3. FUCCI and cell death dynamics upon treatment with THZ531 and TKIs. **A** FUCCI cell cycle progression in Hs578T cells 12, 18, 24, 48 and 72 hours after treatment with lapatinib (3.16 µM), nilotinib, rabusertib, erlotinib, momelotinib and ralimetinib (all 1 µM) alone or in combination with THZ531 (0.1 µM) and single treatment of high dose THZ531 (1 µM) or cisplatin (10 µg/mL). Cdt1-positive cells are in G1 phase, Cdt1- and Geminin-positive cells are stalled at the G1/S checkpoint, Geminin-positive cells are in S, G2 or M-phase and negative cells are in transition from M to G1 phase. **B, C** Induction of (apoptotic) cell death measured by Annexin V and PI staining in Hs578T **(B)** or SKBR7 **(C)** after 12, 18, 24, 48 and 72 hours of treatment with these inhibitors. Cell death and FUCCI data are the mean (±SD) of three independent experiments.

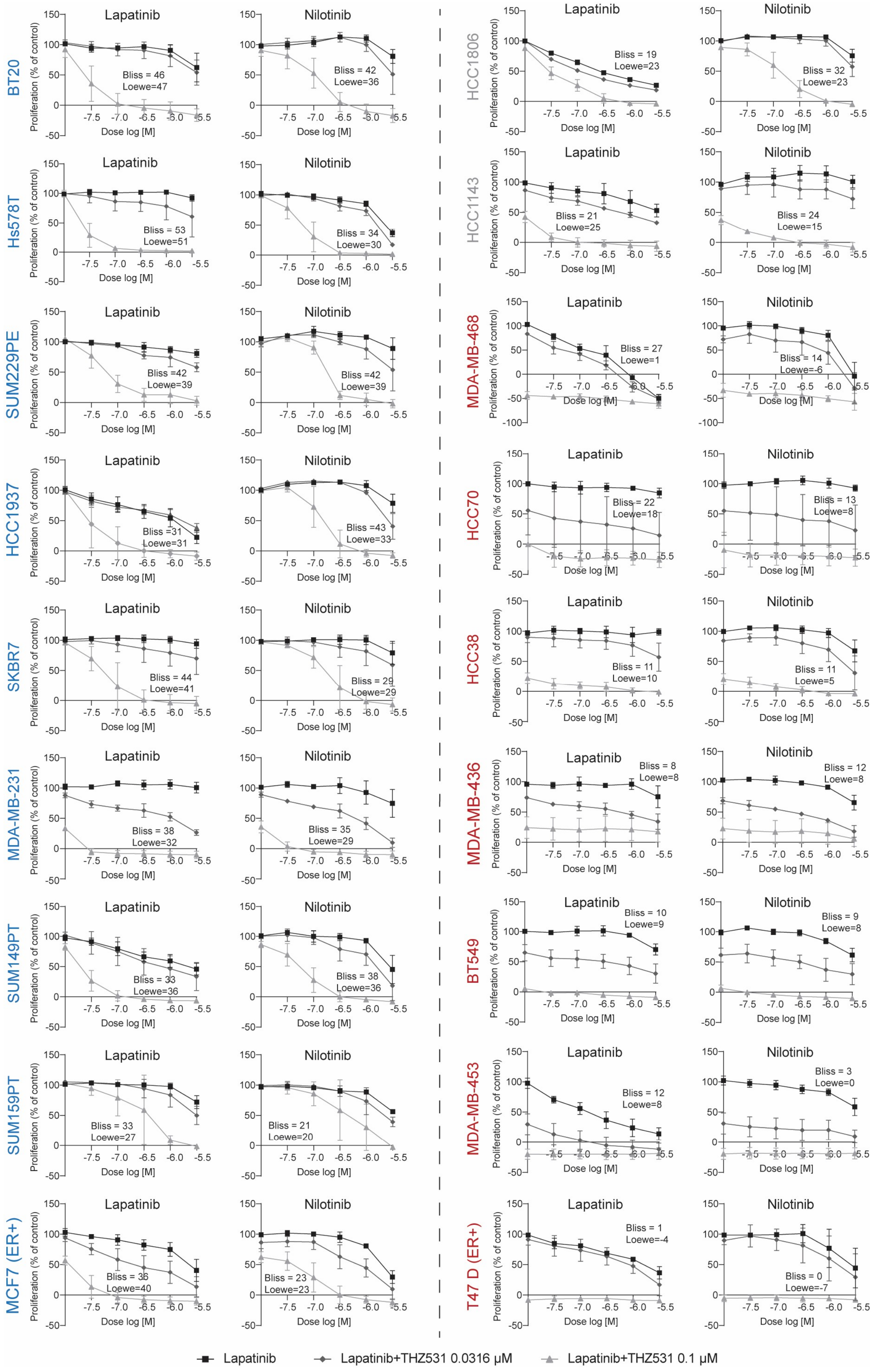

Fig. S4. Dose responses of combination treatment of THZ531 with lapatinib and nilotinib in TNBC cell line panel. Proliferative responses of TNBC cell lines and ER+ BC MCF7 and T47D cell lines to single and combination treatment of lapatinib and nilotinib with THZ531 0.0316 and 0.1 µM. Data were used to calculate BLISS synergy scores. Cell lines coloured in blue were synergistically affected by these combination treatments, while cell lines in red were not. For cell lines indicated in grey only weak synergy or synergy for only one of the compounds was observed. Data are the mean

(±SD) from three independent replicates. For MDA-MB-468, MDA-MB-436 and SUM159PT data are mean (±SD) from two independent replicates.

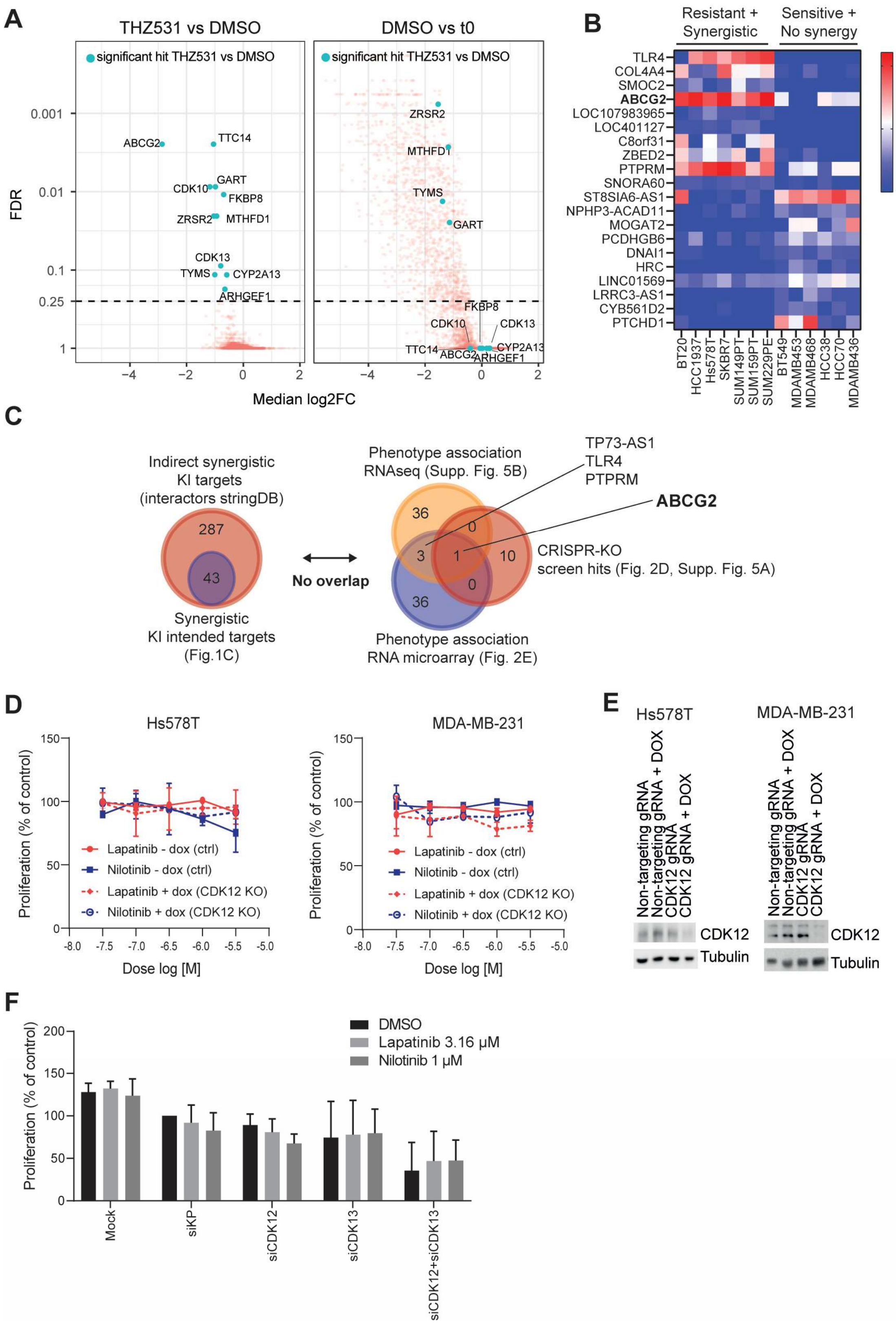

Fig. S5. Pharmacological interaction and ABCG2 are key to synergistic interaction of lapatinib and nilotinib with CDK12/13 inhibitor THZ531. **A** CRISPR-Cas9 dropout screening results showing the Log2 FC of THZ531 versus DMSO of gene knockouts and their effects on proliferation without treatment (DMSO versus T0). Hits that were significantly dropped out upon treatment with THZ531 are highlighted. **B** Rank-ordered RNA sequencing-based mRNA expression from gene set enrichment analysis of THZ531-resistant and synergistic cell lines versus THZ531-sensitive and non-synergistic cell lines showing the top and bottom 10 genes correlated with this phenotype. **C** Lack in overlap between targets of synergistic kinase inhibitors and potential interactors of them (mined using StringDB's experimentally confirmed interactions with confidence score of 0.7) with the top 20 gene associations of the resistance/sensitive phenotype from RNA-sequencing or RNA microarray data, and with significant hits from CRISPR-Cas9 knockout sensitization screening with THZ531 (see

Supplementary Table 2). **D** Proliferative responses of Hs578T (left) and MDA-MB-231 (right) cells with dox-inducible Cas9 and ABCG2 gRNA to treatment with dose range of lapatinib or nilotinib, with and without doxycycline (CDK12 knockout or ctrl). **E** Western blots showing reduced CDK12 protein expression in ABCG2 gRNA expressing Hs578T or MDA-MB-231 cells after addition of doxycycline. **F** Proliferative responses to treatment with lapatinib (3.16 μM) or nilotinib (1 μM) in Hs578T cells with CDK12, CDK13 or CDK12/CDK13 (double) knockdown. Data are the mean (±SD) of three independent experiments or representative (western blot) of three independent experiments.

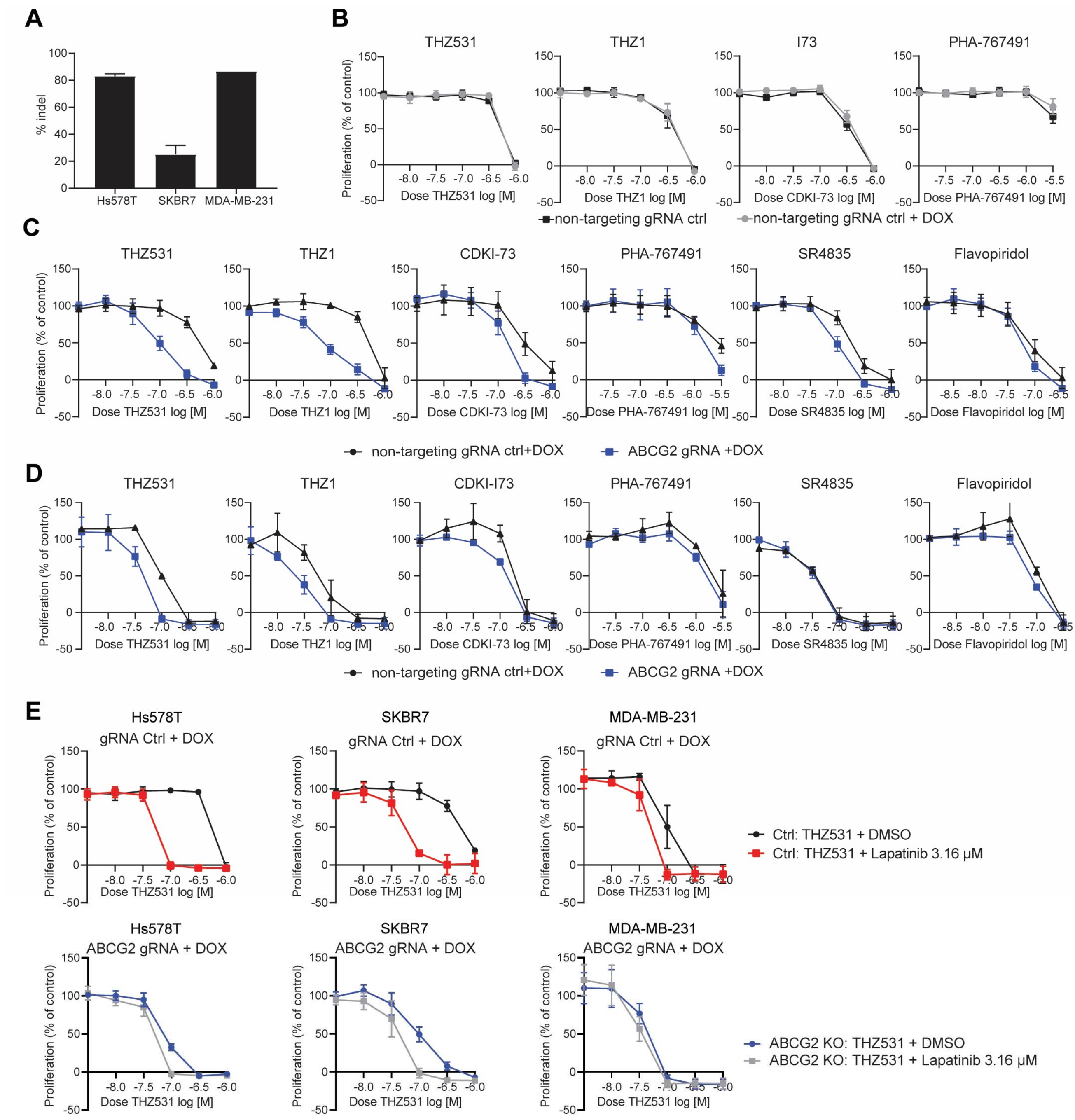

Fig. S6. ABCG2 knockout synergizes with THZ531 in similar fashion as TKIs. **A** Knockout efficiency of ABCG2 4 days after doxycycline induction in Hs578T, SKBR7 and MDA-MB-231 iCas9 cells expressing ABCG2 gRNA, as determined by %indel using TIDE compared to controls without doxycycline induction. Data are mean (±SD) of two independent experiments. **B** Proliferative response to transcriptional CDK inhibitors for Hs578T cells with non-targeting gRNA ctrl with and without doxycycline (DOX) treatment, showing no effect of doxycycline induction itself. **C, D** Proliferative response to transcriptional CDK inhibitors for SKBR7 **(C)** or MDA-MB-231 **(D)** cells expressing ABCG2 gRNA after treatment without doxycycline (Ctrl) or with doxycycline (ABCG2 KO). **E** Effect of combination treatment with lapatinib (3.16 μM) and THZ531 in Hs578t, SKBR7 and MDA-MB-231 in control (above, non-targeting gRNA ctrl induced with doxycycline) versus ABCG2 knockout (below). All proliferation data are the mean (±SD) from three independent experiments.

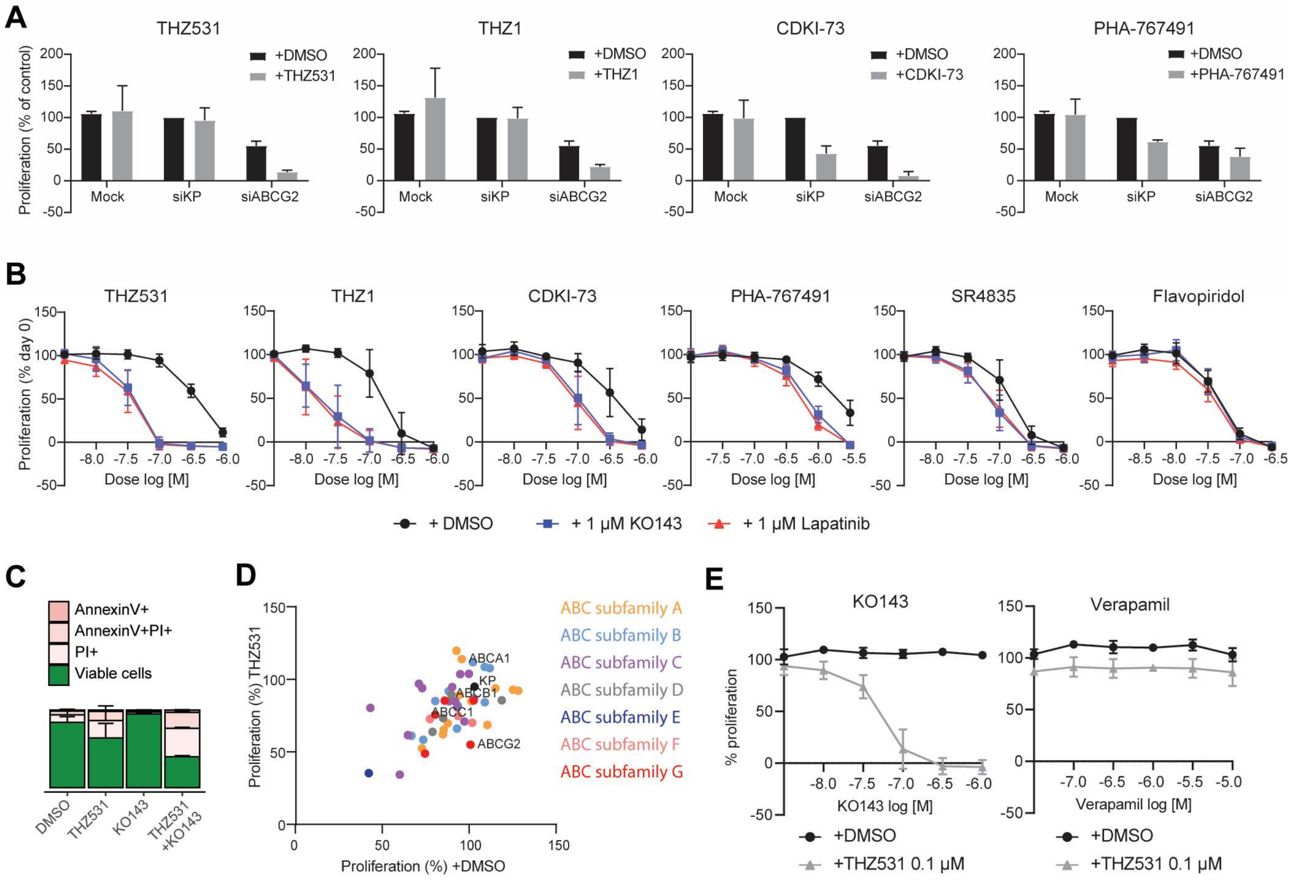

Fig. S7. ABCG2 inhibition and knockdown synergizes with THZ531 in similar fashion as TKIs in SKBR7 cells. **A** Proliferative response to transcriptional CDK inhibitors in SKBR7 cells treated with transfection reagent (Mock), siKP (Ctrl) or siABCG2. **B** Proliferative responses of dose ranges of transcriptional CDK inhibitors alone, or in combination with KO143 (1 µM) or lapatinib (1 µM) in SKBR7 cells. **C** Effects of combination treatment of KO143 (1 µM) with THZ531 (0.1 µM) on AnnexinV/PI stained cell death (lower, 72 hr) in SKBR7 cells. **D** ABC-transporter siRNA screen showing proliferative responses upon knockdown of ABC-transporter genes together with DMSO or THZ531 (0.1 µM) treatment in SKBR7 cells. **E** Proliferative responses of combination treatment of dose range of either ABCG2 inhibitor KO143 or ABCB1/ABCC1 inhibitor Verapamil with THZ531 0.1 µM in SKBR7 cells. All knockdown data are the mean (±SD) from two independent experiments. All data from combination treatments are the mean (±SD) from three independent experiments.

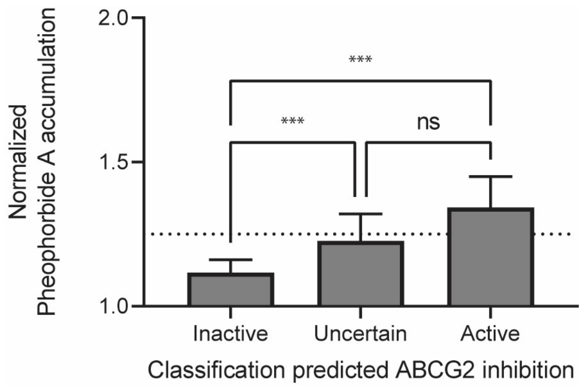

Fig. S8. Pheophorbide A accumulation is higher for compounds predicted to have ABCG2 inhibitory activity. Bar graphs represent the median normalized pheophorbide A accumulation, with 95% confidence intervals. Mean ranks were compared using Kruskal-Wallis testing and corrected for multiple testing using the Benjamini and Hochberg method.

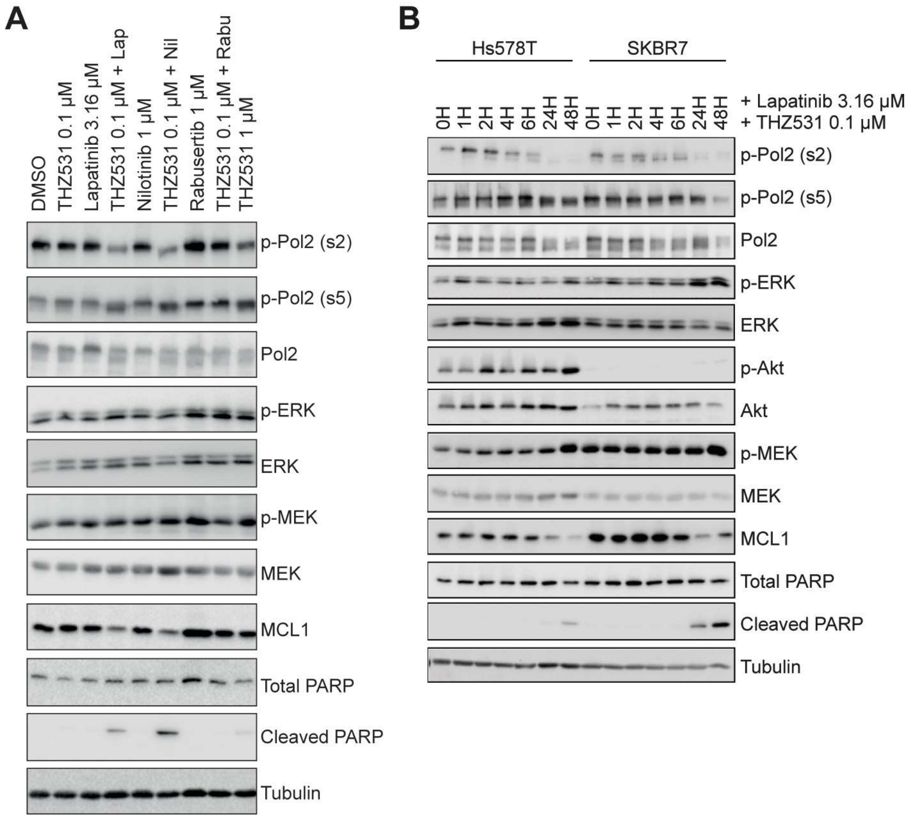

Fig. S9. Protein levels after treatment with kinase inhibitors and THZ531. **A** Western blot showing protein levels of (phosphorylated) C-terminal domain of RNA polymerase II (Pol2), ERK, MEK, MCL1, total and cleaved PARP and tubulin 24 hours after treatment with THZ531 (0.1 µM), lapatinib (3.16 µM), nilotinib (1 µM), rabusertib (1 µM), a combination thereof, or THZ531 (1 µM) in SKBR7 cells. P-Akt levels were not detectable in SKBR7 cells. **B** Western blot showing the levels of (phosphorylated) C-terminal domain of RNA polymerase II (Pol2), ERK, Akt, MEK, MCL1, total and cleaved PARP and tubulin after 0, 1, 2, 4, 6, 24 and 48 hours treatment with the combination treatment of lapatinib (3.16 µM) and THZ531 (0.1 µM) in Hs578T and SKBR7 cells. Western blot images are representative of two independent experiments.

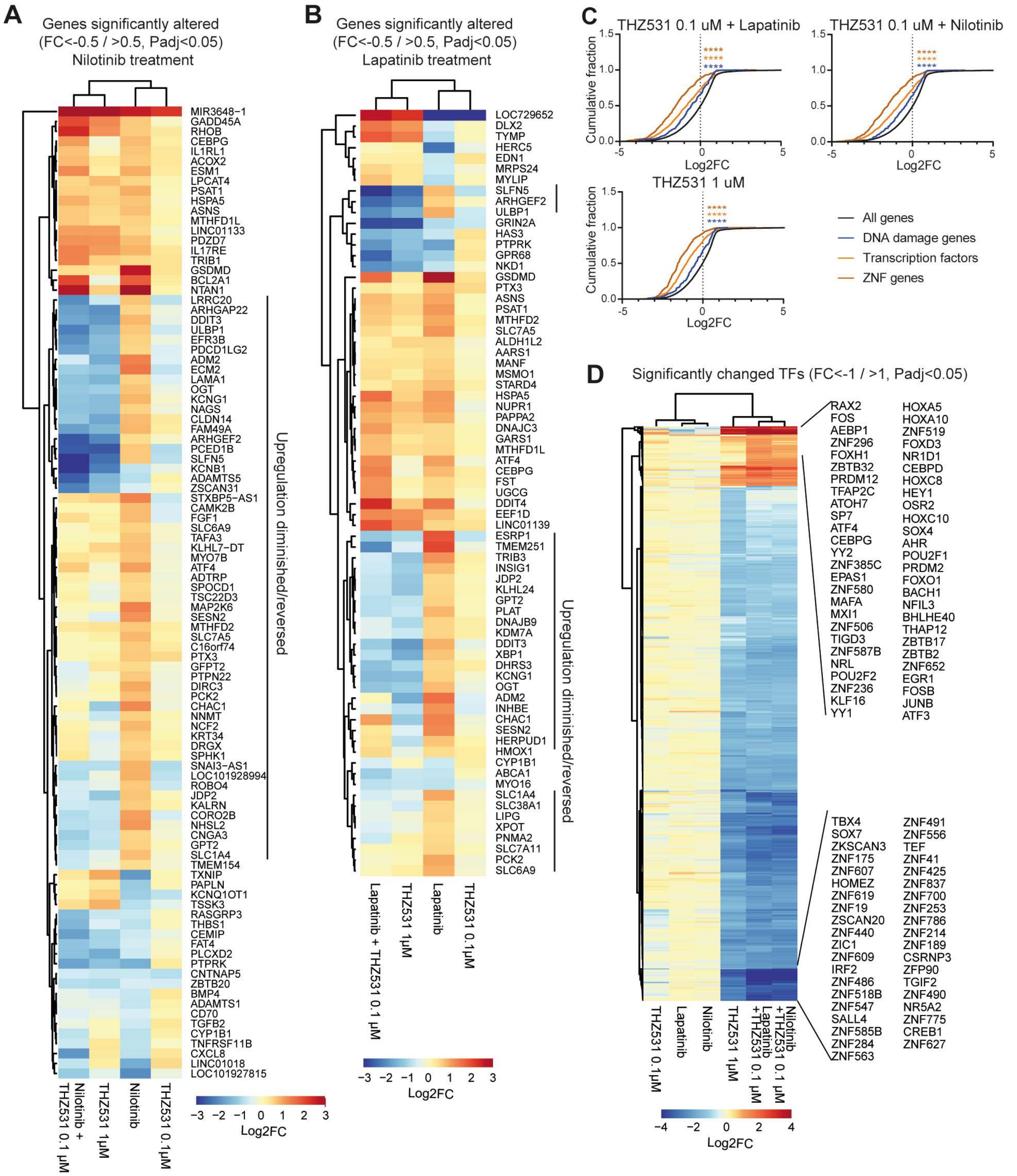

Fig. S10. Differentially expressed genes in Hs578T upon combination treatment with THZ531. **A-B** Log2 FCs of genes significantly up- or downregulated (Log2 FC<-0.5 or >0.5, adjusted *p*<0.05) upon treatment with single nilotinib **(A)** or lapatinib **(B)** treatment. nilotinib or lapatinib upregulated genes that are not as strongly upregulated in the combination treatment are highlighted by the vertical line. **C** Cumulative fraction plots showing gene expression changes of all genes versus (Zinc finger, ZNF) transcription factors or DNA damage genes. Distributions were compared using Kolmogorov-Smirnov tests. **D** Selection of transcription factor genes strongly up- or downregulated (Log2 FC<-1 or >1, adjusted *p*<0.05) in at least one of the treatments.

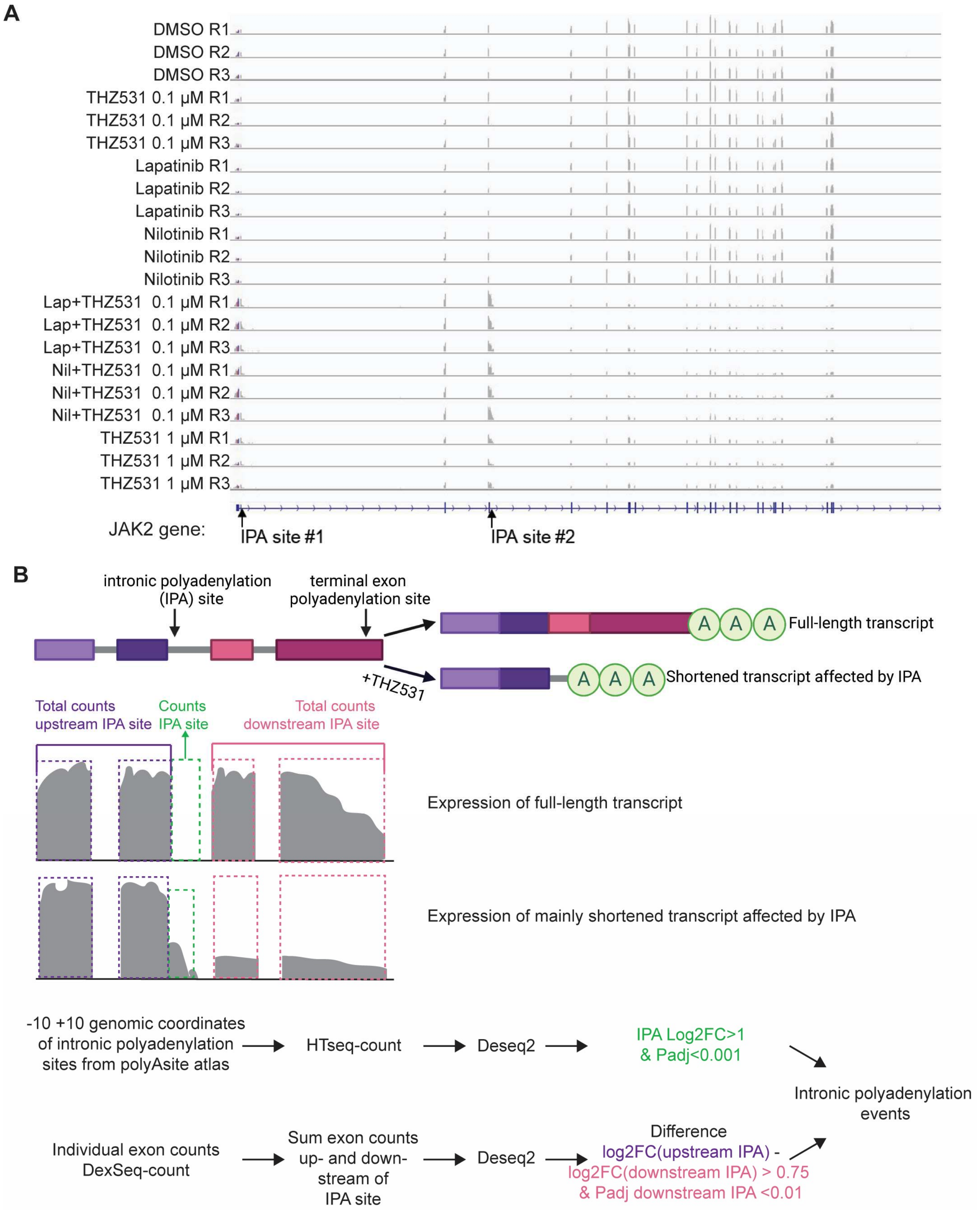

Fig. S11. Analysis of intronic polyadenylation in RNA sequencing data**. A** Example of gene tracks for the JAK2 gene, showing intronic polyadenylation (IPA) for all the independent replicates treated with lapatinib/Nilotinib and THZ531 0.1 µM or high dose THZ531 1 µM. **B** Schematic of IPA and its effect on polyA-enriched sequencing data and sequencing tracks. Possible intronic polyadenylation sites, with polyadenylation signals, are described in the polyAsite atlas and the region of -10 and +10 nucleotides was counted using HTseq-count. Differential expression of these IPA sites was analyzed using Deseq2. Individual exons were counted using DexSeq-count and the differential expression of the sum from all exons upstream (before) of the IPA site and downstream (after) of the IPA site was determined using Deseq2. Intronic polyadenylation events were considered significant when both the IPA site itself was significantly upregulated (Adjusted $p<0.001$, Log2 FC>1) and the difference between the expression of exons before and after the IPA site was larger than 0.75, while the exons after the IPA site were significantly downregulated (Adjusted $p<0.01$).

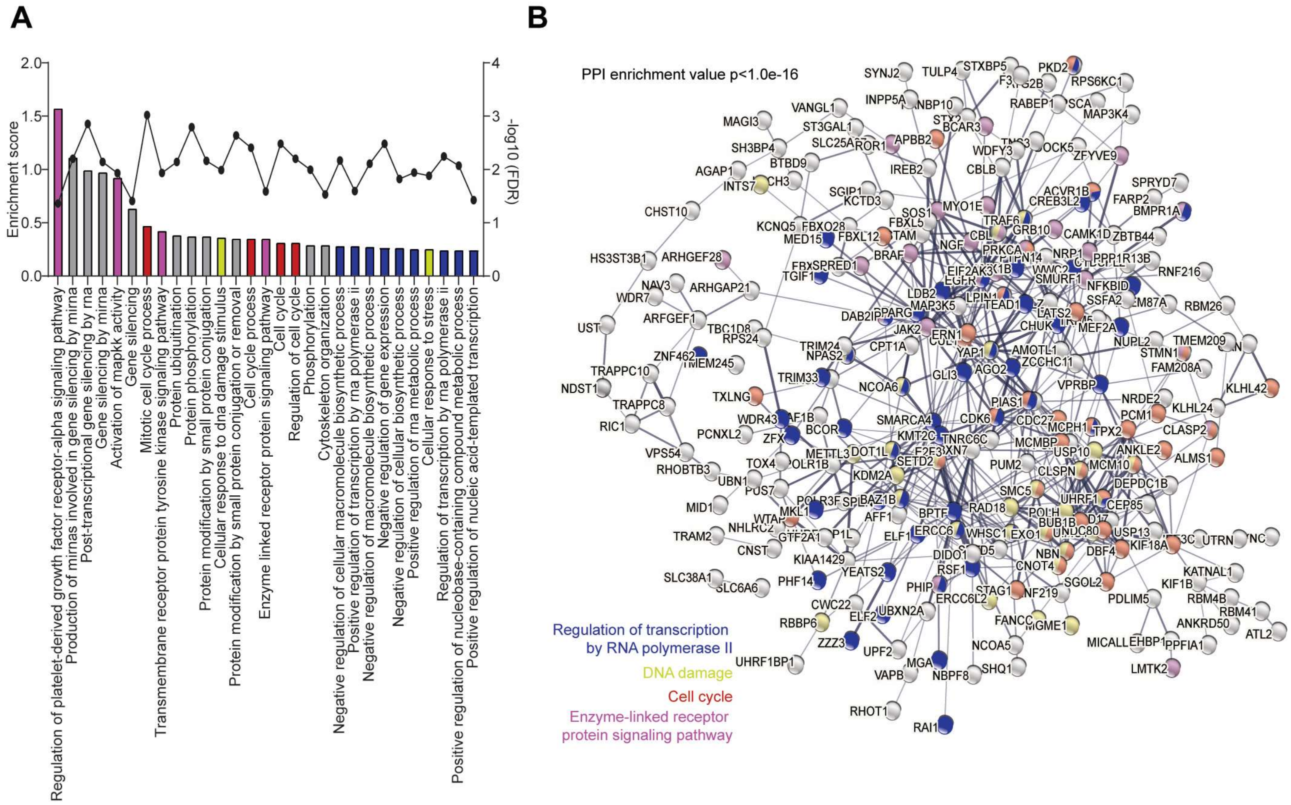

Fig. S12. Pathways affected by IPA after treatment with lapatinib and THZ531. **A** Top 30 significantly enriched pathways (GO biological process) among genes with IPA event upon treatment with lapatinib and THZ531. **B** Gene network showing interactions between genes with IPA event and protein-protein interaction (PPI) enrichment *p* value. Disconnected nodes (62/306) were not shown in the network. Data are from RNA-sequencing analysis performed on samples derived in three independent experiments.

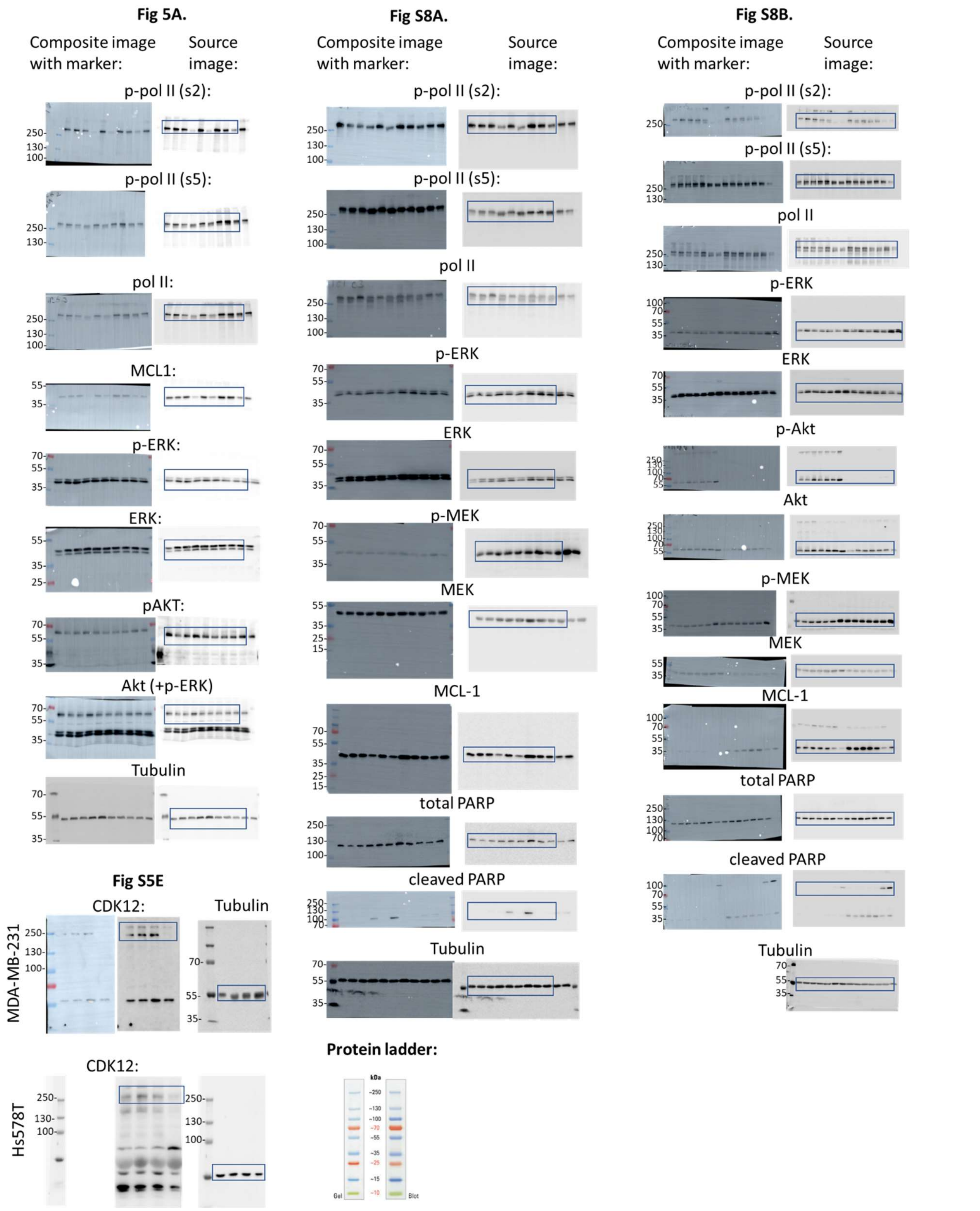

Fig. S13. Uncropped western blot images. Composite images (left) of chemiluminescent or fluorescent (Tubulin) signal with colorimetric signal of the protein ladder, if available. Source images (right) of chemiluminescent signal or fluorescent signal (Tubulin) used for the cropped images in Fig. 5A, S5E, S9A and S9B. Blots were mostly cut into two pieces to stain for small and large proteins. The approximate cropping areas used for the final images are indicated by the blue squares. Lanes not used for the final image represent irrelevant conditions (e.g. additional positive/negative controls).
